# A Phase I, Dose-Finding Study of Sorafenib in Combination with Gemcitabine and Radiation Therapy in Patients with Unresectable Pancreatic Adenocarcinoma: A Grupo Español Multidisciplinario en Cáncer Digestivo (GEMCAD) Study

**DOI:** 10.1371/journal.pone.0082209

**Published:** 2014-01-09

**Authors:** Jorge Aparicio, Carmen García-Mora, Marta Martín, Ma Lourdes Petriz, Jaime Feliu, Ma Elena Sánchez-Santos, Juan Ramón Ayuso, David Fuster, Carlos Conill, Joan Maurel

**Affiliations:** 1 Department of Medical Oncology, Hospital Universitario y Politécnico La Fe, Valencia, Spain; 2 Department of Radiation Oncology, Hospital Universitario y Politécnico La Fe, Valencia, Spain; 3 Department of Medical Oncology, Hospital de Sant Pau, Barcelona, Spain; 4 Department of Radiation Oncology, Hospital de Sant Pau, Barcelona, Spain; 5 Department of Medical Oncology, Hospital Universitario La Paz, Madrid, Spain; 6 Department of Radiation Oncology, Hospital Universitario La Paz, Madrid, Spain; 7 Department of Radiology, Hospital Clínic, Barcelona, Spain; 8 Department of Nuclear Medicine, Hospital Clínic, Barcelona, Spain; 9 Department of Radiation Oncology, Hospital Clínic, Barcelona, Spain; 10 Department of Medical Oncology, Hospital Clínic, Barcelona, Spain; Davidoff Center, Israel

## Abstract

**Purpose:**

Sorafenib, an oral inhibitor of B-raf, VEGFR2, and PDGFR2-beta, acts against pancreatic cancer in preclinical models. Due to the radio-sensitization activity of both sorafenib and gemcitabine, we designed a multicenter, phase I trial to evaluate the safety profile and the recommended dose of this combination used with concomitant radiation therapy.

**Methods:**

Patients with biopsy-proven, unresectable pancreatic adenocarcinoma (based on vascular invasion detected by computed tomography) were treated with gemcitabine (300 mg/m2 i.v. weekly ×5 weeks) concurrently with radiation therapy (45 Gy in 25 fractions) and sorafenib (escalated doses in a 3+3 design, from 200 to 800 mg/day). Radiation portals included the primary tumor but not the regional lymph nodes. Patients with planning target volumes (PTV) over 500 cc were excluded. Cases not progressing during chemoradiation were allowed to continue with sorafenib until disease progression.

**Results:**

Twelve patients were included. Three patients received 200 mg/day, 6 received 400 mg/day, and 3 received 800 mg/day; PTVs ranged from 105 to 500 cc. No dose-limiting toxicities occurred. The most common grade 2 toxicities were fatigue, neutropenia, nausea, and raised serum transaminases. Treatment was discontinued in one patient because of a reversible posterior leukoencephalopathy. There were no treatment-related deaths.

**Conclusion:**

The addition of sorafenib to concurrent gemcitabine and radiation therapy showed a favorable safety profile in unresectable pancreatic adenocarcinoma. A dose of 800 mg/day is recommended for phase II evaluation.

**Trial Registration:**

EudraCT 2007-003211-31 ClinicalTrials.gov 00789763

## Introduction

Pancreatic cancer ranks sixth among cancer-related deaths in Europe. The overall 5-year survival rate is <5%. Less than 20% of patients present with localized, potentially curable tumors, amenable to surgical resection. Approximately 30% of patients with pancreatic cancer receive a diagnosis of advanced locoregional disease, and an additional 30% of patients will have local recurrence of tumors after treatment for early disease. Treatment of patients with locally advanced disease is palliative; with current therapies, the median overall survival ranges only from 9 to 10 months [1]. Management options include systemic chemotherapy alone and combined forms of treatment with chemoradiation and chemotherapy. A series of randomized trials conducted over the past two decades established that chemoradiation therapy is an effective, although more toxic, approach in these patients [2].

Gemcitabine has been considered for many years the standard therapy for metastatic pancreatic cancer. Recently Folfirinox schedule, has replace gemcitabine as first-line therapy in fit (ECOG PS 0–1) metastatic pancreatic cancer patients [3]. Gemcitabine remains a standard drug for locally unresectable pancreatic cancer in combination with radiotherapy. Previous phase I and II studies determined that gemcitabine could be safely combined with radiotherapy directed to the gross disease without inclusion of clinically negative lymph node-bearing areas. Response rates of 20% have been reported [4]–[5]

Sorafenib is an oral inhibitor of B-raf, vascular endothelial receptor 2 (VEGFR2), and PDGFR2-beta that has shown activity against pancreatic cancer using in vivo animal models [6]. Preclinical studies using pancreatic cancer cells have found that gemcitabine is a marked radiation sensitizer, even at noncytotoxic concentrations [7], and that the addition of sorafenib to radiotherapy could enhance tumor growth delay [8]. These findings suggest that the combination of all three agents, gemcitabine, sorafenib, and radiotherapy, could result in improved control of both local and distant sites. The present Phase I study was performed to evaluate the safety profile and to determine the recommended dose of sorafenib in combination with gemcitabine and concomitant radiation therapy.

## Patients and Methods

The protocol for this trial and supporting CONSORT checklist are available as supporting information in Checklist S1 (Annex 1) and Protocol S1 (Annex 2)

Patients with histologically or cytologically proven diagnosis of pancreatic adenocarcinoma were included in the study if they presented a locally advanced disease (judged unresectable by radiological criteria: invasion of celiac trunk or superior mesenteric artery or both), or metastatic disease (with local symptoms amenable for local radiotherapy), or unresectable local relapse (after initial surgery but with no adjuvant therapy). Other inclusion criteria were age >18 years, written informed consent, measurable disease (RECIST criteria), ECOG performance status ≤1, adequate bone marrow, liver, and renal functions (neutrophil count ≥1.5×109/L, platelets ≥100×109/L, hemoglobin ≥9 g/dl, serum bilirubin <1.5× upper limit of normality (ULN), ALT and AST ≤2.5×ULN or ≤5×ULN in case of liver tumor involvement or metastases, amylase and lipase <1.5×ULN, serum creatinine ≤1.5×ULN, creatinine clearance ≥45 ml/min, INR ≤1.5, and APTT and TTP ≤1.5×ULN), and appropriate contraception methods. Main exclusion criteria were prior chemotherapy or radiotherapy, pancreatic surgery in the last 30 days, planned target volume (PTV) for radiotherapy >500 cc, life expectancy <12 weeks, treatment with full dose anticoagulants, unstable cardiopathy or arterial hypertension, HIV+ serology, or active viral hepatitis.

Pretreatment evaluation comprised symptom assessment, physical examination, hematology, serum biochemistry, CA 19.9 levels, coagulation tests, EKG, tumor measurements by means of multidetector or spiral computed tomography (CT) scans, endoscopic ultrasound (EUS), and other symptom-guided explorations when needed. Positron emission tomography (PET)-CT was done before and after treatment in one of the four Institutions. PET Response Criteria in Solid Tumors (PERCIST) was used to evaluate these cases [9]. The planned treatment duration was 5 weeks, and consisted of: a) gemcitabine 300 mg/m2 by 30-minute intravenous infusion on day +1 every week for 5 doses; b) concurrent radiotherapy to a total dose of 45 Gy (1.8 Gy/fraction, 5 times a week for 5 weeks) by means of a linear accelerator, without concomitant boost, and with energies of at least 6 MV with isocentric technique. Radiation portals included the tumor site plus a maximum 2-cm margin and a PTV ≤500 cm3 defined by CT; and c) escalated doses of sorafenib from 200 mg/day to 400 mg twice a day continuously (200 mg tablets). Sorafenib was temporally discontinued when radiotherapy was delayed for more than 3 days. Maintenance with sorafenib was allowed in the absence of tumor progression after chemoradiation.

Dose escalation was performed according to a 3+3 design with three levels: I) 200 mg/day; II) 200 mg twice a day; and III) 400 mg twice a day. Toxicity was reported according to the National Cancer Institute Common Toxicity Criteria, version 3.0. Patients were monitored weekly, including hematology, serum chemistries, and toxicity assessment. Dose reductions and treatment modifications were planned according to specific adverse events. Tumor evaluation by means of CT scans was performed 2 weeks after the end of treatment (week 7). After treatment, patients were evaluated every 2 months until disease progression.

The objective of the study were to assess the safety profile and determine the maximum tolerated dose (MTD), the recommended dose (RD), and the dose-limiting toxicities (DLTs) of sorafenib in combination with gemcitabine and concomitant radiotherapy. If 1 of the 3 patients of the cohort presented DLT, 3 other patients were evaluated at that level. The MTD was defined in a cohort of 3 patients if ≥2 patients presented DLT or in an expanded cohort of 6 patients if ≥3 patients presented DLT. The MTD was projected to be the RD. The DLT was considered as any grade 3 to 4 hematological or nonhematological toxicity producing a stop in radiation therapy for a period of more than 2 weeks, or any treatment-related death. A secondary objective was to explore the overall response (complete + partial) rate by RECIST criteria.

The study was conducted in accordance with the ethical principles of the Declaration of Helsinki and the International Conference on Harmonization of Good Clinical Practice. The protocol was approved by the Reference Ethics Committee (Hospital Clinic Ethics Committee) and the Spanish Medicines Agency (AEMPS). All participating patients provided signed informed consent before enrolment.

## Results

**Figure 1:**
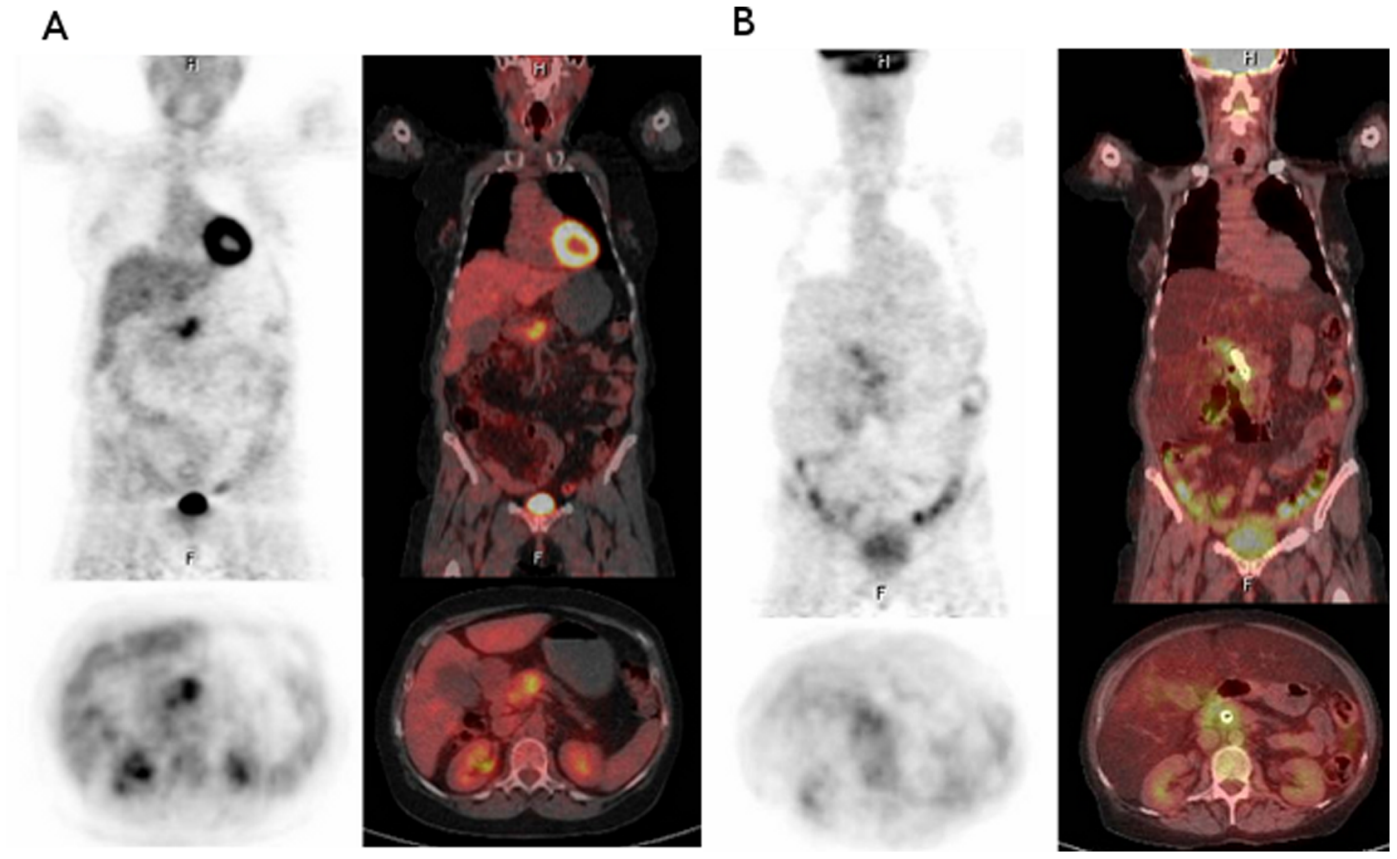
Flowchart of the study. Positron emission tomography-Computed tomography images show pathological uptake (SUVmax = 9.44) in a 4-cm pancreatic mass at baseline (A). There is no evidence of metabolic activity in the follow-up study (B), being considered as a complete response by EORTC criteria (PERCIST criteria).

Twelve patients with unresectable, locally advanced (10) or metastatic (2) pancreatic adenocarcinoma were included between 12/07 and 09/09 (Fig. 1). Median patient age was 59 years, 7 were females, and ECOG performance status was 1 in 11 cases. Main patient characteristics are shown in Table 1. Three patients received 200 mg/day, 6 received 400 mg/day, and 3 received 800 mg/day. Radiation PTVs ranged from 105 to 500 cc. The most common grade 2 toxicities were fatigue (4 cases), neutropenia (3), nausea (5), and raised serum transaminases (4) (Table 2). Treatment was discontinued in one patient at dose level II because of a reversible grade 4 posterior leukoencephalopathy, and was considered a DLT. Three other patients entered level II without additional toxicities. There were no other grade 4 toxicities or hospital admissions and no treatment-related deaths. Grade 3 toxicities were fatigue (1) and thrombocytopenia (1), both cases at dose level III, and the recommended dose was continuous sorafenib 800 mg (400 mg twice a day) in combination with gemcitabine (300 mg/m2) weekly ×5 weeks and radiation therapy (45 Gy in 5 weeks).

**Table 1:** Patient Characteristics.

	n	%
**Age (y)**		
Median	59	
Range	39–69	
**Sex**		
Female	7	58
**ECOG performance status**		
0	1	8
1	11	92
**Tumor location**		
Head	4	33
Body	6	50
Tail	2	17
**Stage**		
Locally advanced	10	
Metastatic	2	
**CA19.9 (U/ml)**		
Median	809	
Range	(7-3561)	
**Weight loss**		
>10%	2	17
**CT arterial invasion**		
Celiac trunk	2	17
Hepatic artery	2	17
Mesenteric artery	6	50
**CT venous invasion**		
Portal vein	4	33
Superior mesenteric vein	8	67
**USE arterial invasion**		
Celiac trunk	1	8
Hepatic artery	0	0
Mesenteric artery	1	8
**USE venous invasion**		
Portal vein	5	42
Superior mesenteric vein	6	50

ECOG: Eastern Cooperative Group.

CT: Computed tomography.

EUS: Endoscopic ultrasound.

**Table 2:** Hematological and nonhematological toxicities.

Grade	II	III	IV	II	III	IV	II	III	IV
Hemoglobin	2	0	0	0	0	0	1	0	0
Leukocytes	3	0	0	4	0	0	2	0	0
Neutrophils	1	0	0	1	0	0	1	0	0
Platelets	0	0	0	0	0	0	0	1	0
Diarrhea	0	0	0	1	0	0	1	0	0
Fatigue	2	0	0	2	0	0	0	1	0
Emesis/vomiting	0	0	0	3	0	0	2	0	0
AST	3	0	0	1	0	0	0	0	0
ALT	2	0	0	1	0	0	1	0	0
Weight loss	0	0	0	1	0	0	0	0	0
Hypertension	0	0	0	1	0	0	0	0	0
Leukoencephalopathy	0	0	0	0	0	1	0	0	0

Toxicity Level I (n = 3) Level II (n = 6) Level III (n = 3).

**Figure 2:**
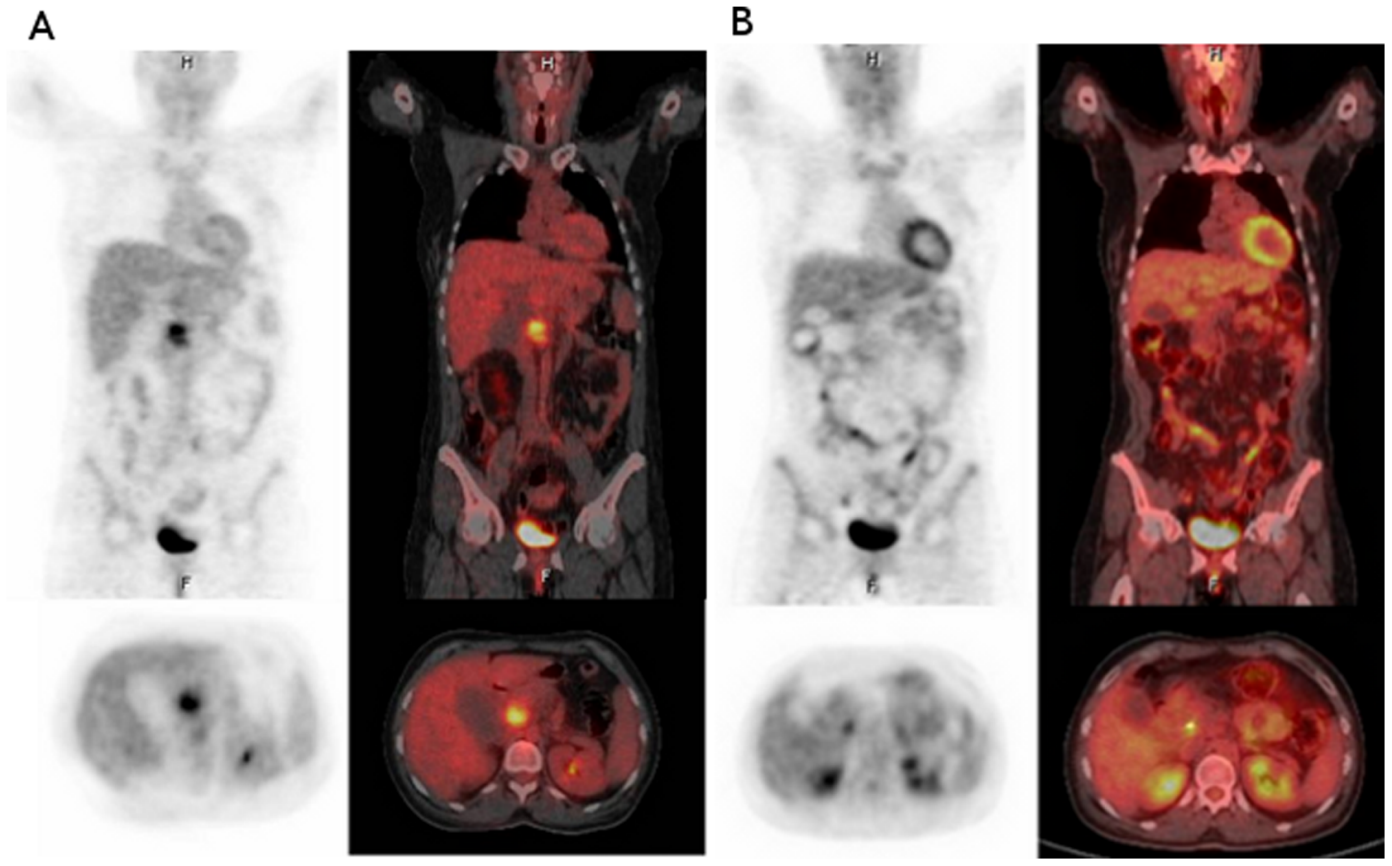
Positron emission tomography-Computed tomography images show pathological uptake (SUVmax = 4.59) in a 20×38 mm mass in the pancreatic body at baseline (A). There is no evidence of metabolic activity in the follow-up study (B), considered a complete response by EORTC criteria (PERCIST criteria).

Eleven patients completed the planned treatment protocol. All patients were evaluable for response. Two partial responses and 7 disease stabilizations were achieved, for an overall response rate of 16.6% and a disease-control rate of 75%. A PET-CT evaluation was done in 5 cases. Following EORTC criteria, 3/5 patients presented CR and 2/5 presented PR. (See Table 3.) Representative cases with CR are shown in Figure 1 and 2. Only two patients underwent surgical resection, one of whom was deemed unresectable at laparotomy. The other patient achieved a partial response by RECIST criteria and underwent a Whipple procedure, but died postoperatively due to anastomotic leak complications. Residual disease was found in the pathological specimen.

**Table 3:** Correlation between positron emission tomography-computed tomography and computed tomography.

Case	Level	PTV (cm3)	CA19.9(basal)U/ml	CA19.9 (post-tt) U/ml	Basal PET-TC	Post-tt PET-TC	Responseby PET-CT	Basal TC (mm)	Post-tt TC (mm)	Response rate (RECIST 1.0)	PFS (months)	Site of progression
1	I	105	533	200	9.44	0	CR	32	25	SD	4	Liver
2	I	234	190	108	2.7	1.8	PR	35	40	SD	10	Local failure
3	II	214	7	11	3.71	2.14	PR	50	39	SD	4	Peritoneum
4	II	333	563	980	4.59	0	CR	38	24	PR	2	Liver
5	III	309	556	733	4.41	0	CR	60	60	SD	5	Peritoneum

Abbreviations:

PTV: Planning target volumes.

Post-tt: Post treatment.

PET: Positron emission tomography.

CT: Computed tomography.

PFS: Progression-free survival.

After a median follow-up of 24 months (range, 15–36), 10 patients have progressed (2 locally, 4 in the liver, 3 in the peritoneum, and 1 in the liver and peritoneum) and 11 have died (including one of upper gastrointestinal bleeding, that occurred 3 months after chemo-radiotherapy treatment, in the absence of tumor progression). Median progression-free and overall survival were 4.4 (95% CI 3.4–5.4) and 10 months (95% CI 5.7–14.3), respectively.

## Discussion

The goals of this phase I trial were to provide an initial safety evaluation of sorafenib added to gemcitabine and radiation in patients with unresectable pancreatic cancer and preliminary assessment of efficacy. Globally the schedule shows a good compliance (11/12 completed the planned therapy with gemcitabine; 91%), with only one case of grade IV toxicity and no toxic-related death. We could not rule out that the astringent radiotherapy PTV volume (<500 cc) selected in our study would probably minimize toxicity. Although DLT was not achieved, we did not plan to escalate up to 800 mg/day, because this is the sorafenib dose approved in advanced settings in hepatocellular carcinoma and renal carcinoma.

There are no previous published clinical data of the combination of sorafenib, radiotherapy and gemcitabine in pancreatic ductal adenocarcinoma (PDAC). Preclinical data of sorafenib combined with radiotherapy suggests that sequential treatment, instead of a concurrent strategy, would be the optimal approach [10]. In hepatocellular carcinoma (HCC) however stereotactic body radiation therapy (SBRT) concomitant with sorafenib 200 mg po was the maximally tolerate dose in high irradiated liver volume (30–60%), due to thrombocytopenia, bowel bled and bowel obstruction [11]. In metastatic pancreas a phase I study shows the feasibility of a combination of capecitabine, oxaliplatina and sorafenib 200 mg BID [12]. Unfortunately recently, a double-blind randomized phase III trial failed to demonstrate any benefit for gemcitabine/sorafenib over gemcitabine/placebo [13].

There are two potential targets for sorafenib efficacy. BRAF is one of the targets, but we know now that in PDAC there is a plethora of intrinsic mechanisms of resistance to BRAF/MEK inhibitors. Theoretically KRAS mutant tumors (90% of patients) would be sensitive to RAF-MEK inhibitors. Unfortunately, additional partially KRAS-independent pathways such as PI3K [14] and STAT-3 [15] and KRAS-dependent pathways such as RAL [16]–[17] and RAC1 [18] can contribute to intrinsic sorafenib and BRAF-MEK inhibitors resistance.

The other target for sorafenib is VEGFR2. Unfortunately, two large randomized trials with more potent agents against VEGFR2 failed to improve results compared to gemcitabine alone in advanced PDAC [19]–[20]. Nonetheless, our clinical data, although preliminary, shows activity in terms of response rate by RECIST criteria (16%) and especially by PERCIST criteria (100%) and local control (75%) that seems at least comparable to gemcitabine and radiotherapy alone [21] or gemcitabine-bevacizumab and radiotherapy [22]. In locally advanced PDAC clinical trials, PET-CT probably could reflect local efficacy more accurately than CT. Sorafenib also inhibits STAT-3 and enhances TRAIL-mediated apoptosis in human pancreatic cell lines [23]. We could not rule out that the association of sorafenib to gemcitabine can increase radiotherapy-induced apoptosis.

A limitation of our study is that systemic therapy probably was insufficient. In fact, most of our patients progressed systemically. It probably would be more valuable to introduce active schedules up front (FOLFIRINOX or gemcitabine-nab-paclitaxel) [24] for 3 months, in order to select the patients who will benefit from local therapies.

In conclusion, the recommended phase II dose for sorafenib in combination with RDT 45 Gy with PTV <500 cc plus weekly 300 mg/m2 gemcitabine, is 400 mg orally BID. In light of the results of the SCALOP trial, a combination with capecitabine plus sorafenib plus radiotherapy could be tested [25]. The addition of nab-paclitaxel to gemcitabine plus radiotherapy, in borderline and locally advanced PDAC is under discussion in the GEMCAD Group.

## Supporting Information

Checklist S1 **TREND Statement Checklist (transparent reporting of trials).**
(PDF)

Protocol S1 **A phase I–II multicenter, non-randomised clinical trial on the safety and efficacy of the combination of sorafenib (bay 43-9006), gemcitabine nd radiation therapy concomitantly in the treatment of patients with locally advanced adenocarcinoma of the pancreas.**
(PDF)
