# Supplementary material for: A Phase I, Dose-Finding Study of Sorafenib in Combination with Gemcitabine and Radiation Therapy in Patients with Unresectable Pancreatic Adenocarcinoma: A Grupo Español Multidisciplinario en Cáncer Digestivo (GEMCAD) Study
Source: PLoS One. 2014 Jan 9;9(1):e82209. doi: 10.1371/journal.pone.0082209 (PMC3886976; doi:10.1371/journal.pone.0082209)
Supplement: Protocol S1 — A phase I–II multicenter, non-randomised clinical trial on the safety and efficacy of the combination of sorafenib (bay 43-9006), gemcitabine nd radiation therapy concomitantly in the treatment of patients with locally advanced adenocarcinoma of the pancreas. (PDF) [file pone.0082209.s002.pdf]

A PHASE I-II MULTICENTER, NON-RANDOMISED CLINICAL TRIAL ON THE SAFETY AND EFFICACY OF THE COMBINATION OF SORAFENIB (BAY 43-9006), GEMCITABINE AND RADIATION THERAPY CONCOMITANTLY IN THE TREATMENT OF PATIENTS WITH LOCALLY ADVANCED ADENOCARCINOMA OF THE PANCREAS

SPONSOR: GEMCAD

Sponsor code: GEMCAD01-07

EudraCT: 2007-003211-31
Investigational new drug: SORAFENIB (BAY 43-9006)

Version 1, 13 June 2007 (final)

CONFIDENTIAL The information and data included in this protocol contain trade secrets and privileged or confidential information. These restrictions will also apply to any information considered as privileged or confidential that is provided in the future. This material can be disclosed and used by the team and collaborators as required for the conduct of the clinical study.

SIGNATURE SHEET

A PHASE I-II MULTICENTER, NON-RANDOMISED CLINICAL TRIAL ON THE SAFETY AND EFFICACY OF THE COMBINATION OF SORAFENIB (BAY 43-9006), GEMCITABINE AND RADIATION THERAPY CONCOMITANTLY IN THE TREATMENT OF PATIENTS WITH LOCALLY ADVANCED ADENOCARCINOMA OF THE PANCREAS

SPONSOR CODE: GEMCAD01-07

EudraCT: **2007-003211-31**

INVESTIGATIONAL NEW DRUG: SORAFENIB (BAY 43-9006)

SPONSOR

GRUPO GEMCAD (Grupo Español Multidisciplinario del Cáncer Digestivo)
Ronda General Mitre, 200, 2º 1ª
08006 Barcelona

COORDINATING INVESTIGATOR

Dr. Joan Maurel
Department of Oncology
Hospital Clinic i Provincial de Barcelona
Calle Villarroel, 170
08036 Barcelona

____________________	____________________
Signature	Date

1.- *SUMMARY*

1.1 Type of application

First clinical trial in a new indication.

1.2 Sponsor identification

GEMCAD (Grupo Español Multidisciplinario del Cáncer Digestivo)
Ronda General Mitre, 200, 2º 1ª
08006 Barcelona

1.3 Study title

A phase I-II multicenter, non-randomised clinical trial on the safety and efficacy of the combination of sorafenib (BAY 43-9006), gemcitabine and radiation therapy concomitantly in the treatment of patients with locally advanced adenocarcinoma of the pancreas.

1.4 Protocol code

Code: GEMCAD 07-01

EudraCT no.: 2007-003211-31

1.5 Principal and coordinating investigator

Dr. Joan Maurel
Department of Oncology
Hospital Clinic i Provincial de Barcelona
Calle Villarroel, 170
08036 Barcelona

1.6 Centres where it is planned to perform the research study and trial investigators at each centre

The list of investigators and participating centres will be enclosed as an appendix.

ANNEX I. Investigators and participating centres.

1.7 Clinical Research Ethics Committees approving the study

The clinical trial protocol will be submitted to the relevant CRECs, as laid down in Royal Decree 223/2004.

1.8 Monitor

CRO PIVOTAL will be responsible for monitoring the trial.

1.9 Name and qualification of the individual authorised by the sponsor to sign the protocol and its amendments

The individuals authorised to sign the protocol and the possible amendments are the sponsor and the coordinating investigator.

1.10 Medical experts of the sponsor responsible for the trial

The responsible medical experts are the principal investigators of each centre specified in section 1.6 of this protocol.

1.11 Experimental drugs and description of treatments

PHASE I

The patients included in this phase will receive the following combination at each dosage level investigated **during five consecutive weeks:**

Level 1:

- Sorafenib 200 mg daily administered po continuously.
- Gemcitabine 300 mg/m2/week in 30-minute iv infusion on Day +1 of each week.
- Radiation therapy on Day +1 to Day +5 of each week 1.8 Gy daily (1.8 Gy x 5 fractions x 5 weeks = 45Gy).

Level 2:

- Sorafenib 200 mg bid administered po continuously.
- Gemcitabine 300 mg/m2/week in 30-minute iv infusion on Day +1 of each week.
- Radiation therapy on Day +1 to Day +5 of each week 1.8 Gy daily (1.8 Gy x 5 fractions x 5 weeks = 45Gy).

Level 3:

- Sorafenib 400 mg bid administered po continuously.
- Gemcitabine 300 mg/m2/week in 30-minute iv infusion on Day +1 of each week.
- Radiation therapy on Day +1 to Day +5 of each week 1.8 Gy daily (1.8 Gy x 5 fractions x 5 weeks = 45Gy).

Table 1. Chart of phase I treatment.

Treatment duration	Weeks	Gemcitabine	Sorafenib	Radiation therapy
**5 weeks receiving gemcitabine+sorafenib+radiation therapy**	Week 1	Day 1	Daily	Days 1, 2, 3, 4 and 5
	Week 2	Day 1	Daily	Days 1, 2, 3, 4 and 5
	Week 3	Day 1	Daily	Days 1, 2, 3, 4 and 5
	Week 4	Day 1	Daily	Days 1, 2, 3, 4 and 5
	Week 5	Day 1	Daily	Days 1, 2, 3, 4 and 5

A maximum of 5 doses of gemcitabine will be administered. In case radiation therapy is delayed, the planned dose of gemcitabine will be continued to a maximum of 5 doses, continuing with sorafenib. Sorafenib will be discontinued when radiation therapy is delayed for over 3 days, and will be reinstituted when radiation therapy is restarted.
Treatment with sorafenib will be maintained until any of the criteria described in Section 6.3 of this protocol *“End of the study treatment”* is met.

PHASE II

The treatment is divided into three stages:

Induction therapy:

The patients will receive the combination of gemcitabine and sorafenib **for 8 weeks :**

- Sorafenib at the RD established in the phase I study with continuous daily administration

- Gemcitabine 1000 mg/m2/week in 30-minute iv infusion for three consecutive weeks followed by a rest week for two cycles (one cycle= 4 weeks).

At the end of the induction treatment, a CT will be performed to evaluate the disease progression. Patients with tumoural regression will discontinue the treatment and will leave the study. In the absence of tumoural progression, the patient will continue treatment in the next steps.

Combination therapy:

The patients will receive the combination of gemcitabine, sorafenib and radiation therapy **for 5 consecutive weeks :**

- Sorafenib at the RD established in the phase I study with continuous daily administration
- Gemcitabine 300 mg/m2/week in 30-minute iv infusion on Day +1 of each week.
- Radiation therapy on Day +1 to Day +5 of each week 1.8 Gy daily (1.8 Gy x 5 fractions x 5 weeks = 45Gy).

Maintenance therapy:

The patients will receive maintenance sorafenib until the end of the clinical trial or tumoural progression:

- Sorafenib 400 mg bid administered po until disease progression.

Table 2. Chart of phase II treatment.

Treatment duration	Weeks	Gemcitabine	Sorafenib	Radiation therapy
**Induction**<br>Treatment with gemcitabine (2 months) + sorafenib	Week 1	Day 1	Daily	
	Week 2	Day 1	Daily	
	Week 3	Day 1	Daily	
	Week 4	Rest	Daily	
	Week 5	Day 1	Daily	
	Week 6	Day 1	Daily	
	Week 7	Day 1	Daily	
	Week 8	Rest	Daily	
**Combination therapy**<br>5 weeks on gemcitabine + sorafenib (RD	Week 9	Day 1	Daily	Days 1, 2, 3, 4 and 5

Phase I) + radiation therapy	Week 10	Day 1	Daily	Days 1, 2, 3, 4 and 5
	Week 11	Day 1	Daily	Days 1, 2, 3, 4 and 5
	Week 12	Day 1	Daily	Days 1, 2, 3, 4 and 5
	Week 13	Day 1	Daily	Days 1, 2, 3, 4 and 5

**Maintenance**<br>Treatment with sorafenib 400 mg bid	Week 14		Daily	
	Week 15		Daily	
	Week 16		Daily	
	Week 17		Daily	
	Week 18		Daily	
	Week 19		Daily	
	Week 20		Daily	
	Week 21		Daily	
	Week 22		Daily	
	…		Daily	

Based on the efficacy and toxicity profile of phase I, the study sponsor will re-assess the suitability of continuing with phase II or closing the study.

1.12 Clinical trial phase

Phase I-II study.

1.13 Study objectives

PHASE I

Primary objective

To assess the safety profile and establish the maximum tolerated dose (MTD) / recommended dose (RD) of sorafenib in combination with gemcitabine and radiation therapy concomitantly.

Secondary objectives

- Percent response (PR/CR) by RECIST criteria.

PHASE II

Primary objective

To establish the antitumoural activity profile assessed as the percentage of progression-free rate (PFR) at 6 months, of the combination of sorafenib, gemcitabine and radiation therapy concomitantly in patients with locally advanced adenocarcinoma of the pancreas not receiving previous treatment.

Secondary objectives

- Percent response (PR/CR) by RECIST criteria.
- Overall survival.
- Toxicity occurring using the National Cancer Institute toxicity criteria (NCI CTC) version 3.0.
- Patients amenable to surgery after treatment

1.14 Design

A Phase I-II multicenter, open, non-randomised clinical trial.

1.15 Disease under study

Patients diagnosed of locally advanced or metastatic adenocarcinoma of the pancreas in Phase I of the study and in Phase II patients with locally advanced adenocarcinoma of the pancreas not previously treated.

1.16 Endpoints

PHASE I

Primary endpoint

- Assessment of toxicity occurring in the cohorts of patients using the toxicity criteria of the National Cancer Institute (NCI CTC) version 3.0.

- Definition of maximum tolerated dose (MTD) and recommended dose.

- Definition of dose-limiting toxicity (DLT).

PHASE II

Primary endpoint

- Percentage of progression-free rate (PFR) at 6 months.

Secondary endpoints

- Percent response by RECIST criteria.
- Overall survival
- Toxicity occurring using the NCI CTC criteria, version 3.0
- Percentage of patients resected (R0) after treatment.

1.17 Study population and total number of patients

PHASE I

This phase will include patients with adenocarcinoma of the pancreas locally advanced or with metastatic disease.
The population to be included will be given by the number of cohorts required to find the recommended dose (RD) according to a dose escalation design consisting in cohorts of 3-6 patients.
If the recommended dose for phase II of the study was below 400 mg, the study sponsor will re-evaluate the suitability of continuing with Phase II or closing the study.

PHASE II

This phase will include patients with locally advanced adenocarcinoma of the pancreas.
Thirty-nine patients with a diagnosis of locally advanced adenocarcinoma of the pancreas not receiving treatment previously must be included.
Eligible patients will give their consent to participate in the study, before performing any specific procedure of it.

1.18. Treatment duration

PHASE I

A duration according to the times established for patient inclusion in each cohort until the recommended dose is found is planned. Subsequently, treatment with sorafenib will be maintained until any of the criteria described in Section 6.3 of this protocol *"End of the study treatment"* is met.

PHASE II

The planned treatment administration including the abovementioned three phases (induction, combination with concomitant radiation therapy and maintenance) until disease progression, appearance of toxicity that cannot be managed with the dose changes established in the protocol, or until decided by the investigator or refusal of the patient to continue with the proposed treatment.
After the end of the study treatment, the patients will be included in the follow-up period and contacted regularly (every 2 months) and followed until the death or closure of the study to establish the date of disease progression (if this has not occurred yet) and/or death. The subsequent treatments received will be recorded in the case report form (CRF).

1.19 Timetable and planned ending date

Recruitment period: 12 months for Phase I and 12 months for Phase II from inclusion of the first patient.
Follow-up period: 6 months after inclusion of the last patient

Planned duration of the study: 30 months

2.- CONTENTS

1.- SUMMARY 3

2.- CONTENTS 11

3.- GENERAL INFORMATION 13

4.- RATIONALE AND OBJECTIVES OF THE STUDY 16

5.- CLINICAL TRIAL TYPE AND DESIGN 21

6.- SUBJECT SCREENING 28

7.- DESCRIPTION OF TREATMENT 33

8.- ASSESSMENT CRITERIA AND STUDY CONDUCT 42

9.- SAFETY. REGISTRATION AND REPORTING PROCEDURE 47

10.- ETHICS 52

11.- PRACTICAL CONSIDERATIONS 54

12.- STATISTICAL ISSUES 58

13.- REFERENCES 63

14.- ANNEX I. INVESTIGATORS AND PARTICIPATING CENTRES 67

15.- ANNEX II. CLINICAL RESEARCH ETHICS COMMITTEES EVALUATING THE STUDY 68

16.- ANNEX III. CASE REPORT FORM 69

17.- ANNEX IV INVESTIGATOR'S BROCHURE 70

18.- ANNEX V. STANDARD OPERATING PROCEDURES 71

19.- ANNEX VI. DEFINITIONS 72

20.- ANNEX VII. DECLARATION OF HELSINKI 78

21.- ANNEX VIII. SAE REPORT FORM 79

22.- ANNEX IX. INSTRUCTIONS FOR PERFORMING THE CT 80

23.- ANNEX X. COMMITMENT OF THE INVESTIGATORS 83

3.- GENERAL INFORMATION

3.1 Identification of the trial

3.1.1 Code

Code: GEMCAD01-07

EudraCT no.: 2007-003211-31

3.1.2 Title

A Phase I-II multicenter, non-randomised clinical trial on the safety and efficacy of the combination of sorafenib (Bay 43-9006), gemcitabine and radiation therapy concomitantly in the treatment of patients with locally advanced adenocarcinoma of the pancreas.

3.2 Type of clinical trial

A Phase I-II multicenter, open, non-randomised clinical trial.

3.3 Description of the study products

The full information about the study drugs is included in the enclosed data sheets: Gemcitabine and in the investigator's brochure of (BAY 43-9006) Sorafenib.

3.4 Sponsor data

GEMCAD (Spanish Multidisciplinary Group of Gastric Cancer)

3.5 Monitor identification

CRO PIVOTAL
C/ Gobelas 19 Madrid 28023
Tel.: 91 708 12 50
Fax: 91 708 13 08
C/ Entença 332-334 7º 4º
08029 Barcelona
Tel.: 93 444 17 47
Fax: 93 444 17 46

3.6 Data on trial investigators

Trial coordinator:

Dr. Joan Maurel
Department of Oncology
Hospital Clinic i Provincial de Barcelona
Calle Villarroel, 170, 08036 Barcelona

3.7 Study sites

The list of investigators and participating centres will be enclosed as an appendix.

ANNEX I. Investigators and participating centres.

3.8 Planned duration of the trial

Recruitment period: 12 months for Phase I and 12 months for Phase II from inclusion of the first patient.
Follow-up period: 6 months after inclusion of the last patient

Planned duration of the study: 30 months

4.- *RATIONALE AND OBJECTIVES OF THE STUDY*

4.1 Introduction

Adenocarcinoma of the pancreas is a malignancy that occurs in the pancreatic ductal epithelium. In most cases, the tumour is located in the head of the pancreas, while only in 25% it affects the body or tail.

It is the sixth cause of death from cancer in both women and men in the European Union. The incidence in our country is about 8 cases per 100,000 inhabitants/year. (1)

Surgical resection is the only potentially healing treatment, and it is difficult to perform because at the time of diagnosis, 80-90% of the patients suffer a non-operable tumour, either for its local-regional involvement (30-40%) or for having distant metastasis (50%). In addition, since 80-90% of the patients with localised tumours managed surgically will relapse, it can be stated that at the time of diagnosis or after relapse 90-95% of the patients with adenocarcinoma of the pancreas will be candidate to receive a palliative treatment with chemotherapy. (2)

Survival is very limited, with a rate below 10% at 5 years in patients with regional nodal involvement, and of 25-30% at 5 years in patients undergoing resection surgery. (3)

4.1.1 Treatment of unresectable adenocarcinoma of the pancreas

The TNM classification (Annex VI) is most commonly used to established tumour extent and the criteria of tumour resectability. However, from a therapeutic viewpoint, the classification defining as locally advanced disease that with evidence of surrounding vascular involvement is most commonly used (upper mesenteric artery, celiac trunk, hepatic artery or close >15mm in the porto-mesenteric junction) and metastatic disease with distant involvement.

Of 80% of the patients with unresectable disease on diagnosis, about a half have a locally advanced disease and the other half metastatic disease. The median survival of the former is estimated to be around 6-10 months, while for the latter it is only 3-6 months. (4)

For patients with locally advanced disease, chemotherapy treatment alone or in combination with radiation therapy is the therapeutic option. The addition of chemotherapy to radiation therapy appears to increase its local effects and provides a therapeutic effect for the disease out of the field of radiation.

There cost-efficacy analysis (5) favourable to the active treatment, but it considers that concomitant radiation therapy-chemotherapy improve quality of life through the control of symptoms and prolongs mean survival (10 vs 4.4 months).
The best combination regimen of drugs and radiation therapy is to be defined. With this regard, the results of the clinical trial have not been consistent.
The cytostatics most commonly used are 5-fluorouracil, gemcitabine, cisplatin or paclitaxel.(6)

4.1.2 Chemotherapy-radiation therapy with gemcitabine

Gemcitabine is a deoxycytidine analogue nucleoside metabolised intracellularly to disphosphate (dFdCDP) and triphosphate nucleoside (dFdCTP), both with cytostatic activity by inhibition of the synthesis of DNA (by inhibition of reductase ribonucleotide and the competition with other nucleosides in the addition to DNA), which appears to induce the programmed cell death process called apoptosis. Furthermore, a very small amount of gemcitabine can be also added to the RNA.
Its mechanism of antitumoural action is specific of the cellular phase, mainly by the destruction of cells that are synthesizing DNA (phase-S) and under some conditions blocking the progression of cells that are in phases G1 and S. (7)
Gemcitabine shows a fast plasma clearance, mainly to the inactive metabolite 2'-deoxy-2',2' difluorouridine (dFdU). Less than 10% of an intravenous dose is recovered in urine as unchanged gemcitabine.
Plasma protein binding is negligible. For the recommended dosage regimen, gemcitabine clearance should be virtually completed between 5 and 11 hours from the start of infusion. Gemcitabine does not build up when administered once a week.
Systemic clearance ranged from about 30 $l/h/m^2$ to 90 $l/h/m^2$ depending on the sex and age (interindividual variability of 52.2%).
The clearance of the inactive metabolite dFdU depends on renal excretion and can accumulate if renal function is reduced. Studies have been performed in patients with moderate renal or liver insufficiency, without requiring a special dose adjustment. No data are available for severe insufficiencies.
Gemcitabine is indicated in the treatment of patients with locally advanced or metastatic adenocarcinoma of the pancreas. It is also indicated in patients with cancer of the pancreas refractory to 5-fluorouracil. (8)
The use of gemcitabine as chemotherapeutic agent in adenocarcinoma of the pancreas provides an improvement in the symptoms related to the disease (clinical benefit) and a slight survival advantage. (9)

Studies in vitro suggest that gemcitabine is a potent radiosensitising agent and one of its mechanisms of action consists of reducing the threshold for cell apoptosis induced by radiation. (10-12) Studies performed suggest that radiation therapy and low doses of gemcitabine can be administered concomitantly (administration interval ≤ 7 days) with an acceptable toxicity.
A Phase II trial in patients with locally advanced adenocarcinoma of the pancreas on a weekly gemcitabine regimen (600 mg/m2) and concomitant radiation therapy (50.4 Gy) followed by gemcitabine in single therapy evidenced an overall response rate of 21% and stable disease in 57%. (13) Of the 28 patients included in the study, two could undergo tumoural resection after treatment. The median survival was 7.9 months (range 6.6-17.1) with 1-year survival of 31.1%.

The group of MD Anderson published a Phase I trial aimed at finding the recommended dose of gemcitabine administered weekly (escalated from 350 mg/m2 to 500 mg/m2 weekly x 7 weeks) together with hypo-fractionated radiation therapy (3000 cGy in 10 fractions).(14) The recommended dose was 350 mg/m2 and partial responses were evidenced in 20% of the patients. One-year survival in patients with objective responses was 66%.

4.1.3 Sorafenib in the treatment of adenocarcinoma of the pancreas

Sorafenib is a novel potent diaryl-urea available for oral administration, with a double anti-cancer action by inhibiting proliferation of tumour cells and tumour angiogenesis. The target of sorafenib is the signalling pathway in the Ras/Raf/MEK/ERK cascade in the kinase Raf, and, by its inhibitory effects on this cascade of myogenic kinases, it interferes with cell proliferation, differentiation and survival. (15) It also acts on proangiogenic receptors.
Preclinical trials have shown that sorafenib inhibits the following targets in vitro: (16)

- Raf-1
- Wild-type B-Raf.
- Oncogenic b-Raf V600E
- Vascular endothelium growth factor receptors (VEGFR1,2 and 3), platelet-derived growth factor â receptor (PDGFR- â) and RET tyrosine kinase.
- Tyrosine kinase receptors c-Kit and Flt-3 enhancing the development of malignancies.

In animal in vivo models, sorafenib induced the dose-dependent inhibition of tumour growth in several types of tumours (kidney, breast, pancreas, ovary colon and non-small cell carcinoma of the lung).

Four Phase I studies designed for measuring the MTD (17-20) included patients with metastatic solid tumours, resistant to treatment and progressing (n=173). The MTD and the optimum treatment regimen were established in continued oral administration of 400 mg bid.

As with many other neoplasms, the origin of malignant tumoural proliferation in adenocarcinoma of the pancreas is related to the activation of proto-oncogenes and the inactivation of suppressing genes. One of the families of oncogenes directly involved in adenocarcinoma of the pancreas is the Ras family that in turn activates the Raf path of kinases regulating the phosphorylation of multiple transcription factors with a critical role in cell proliferation, differentiation and survival. (21,22)

The vascular endothelium growth factor (VEGF) plays a major role in tumoural angiogenesis and subsequent dissemination of adenocarcinoma of the pancreas. (23-25) In animal models, the VEGF receptor inhibitors and anti-VEGF antibodies inhibited tumoural growth and angiogenesis and enhanced the antitumoural effect of gemcitabine. (26,27)

A Phase II study (28) recently reported evaluated the efficacy of the combination of sorafenib and gemcitabine as first-line (sorafenib 400 mg bid continuously and gemcitabine 1000 mg/m2 in 30-minute infusion administered on Days +1, +8 and +15 every 28 days). Although the combination was well tolerated, with a toxicity that was mainly haematological (29% grade 3-4 neutropenia, with no episodes of febrile neutropenia) the results were modest without evidencing responses (23% stable disease) and with a 6-month survival of 23% of the patients.

The combination of VEGF inhibitors and radiation therapy is justified as the interference with angiogenic activity can enhance radiosensitisation of tumoural cells in animal models. (29-32) Studies in vitro in melanoma cell lines of the combination of sorafenib with radiation therapy recently reported (33) have shown that this acts as radiosensitising agent assuring this combination for future clinical trials.

4.1.4 Combinations of chemotherapeutic agents with sorafenib

In clinical trials, sorafenib has been administered with other anticancer agents –such as gemcitabine, oxaliplatin, doxorubicin and irinotecan –with its standard dosage regimen. (34)

Sorafenib had no effect on the pharmacokinetics of gemcitabine or oxaliplatin.
Concomitant treatment with sorafenib resulted in an increase of 21% in the AUC of doxorubicin.
When administered with irinotecan, which active metabolite, SN-38, is again metabolised by the UGT1A1, an increase of 67-120% was seen in the AUC of SN-38 and of 26-42% in the AUC of irinotecan.

4.1.5 Rationale for the study

The above data evidence the need for new therapeutic approaches for unresectable adenocarcinoma of the pancreas.
Currently, local control is the prevalent challenge in the treatment of locally advanced disease for the purpose of inducing a morphological remission effect and vascular margin that is more favourable for considering a subsequent tumoural resection.
Although the combination of radiation-chemotherapy plays a major role in the management of inoperable disease and improves the quality of life of patients, the best combination regimen of drugs and radiation therapy is still to be defined.
The disorders enhancing the activation of the family of oncogenes Ras/Raf and abnormalities in the expression of growth factors, including the VEGF, are etiopathogenetic mechanisms described in the adenocarcinoma of the pancreas.
Sorafenib is a small bioavailable molecule with double antitumoural action inhibiting cell proliferation and angiogenesis. Its targets includes the Raf/MEK/ERK path and proangiogenic tyrosine kinase receptors (VEGFR, PDGFR-á and â, RET) in addition to c-KIT and FLT-3, that has been shown to be active in several malignancies, with renal cell carcinoma being the condition where its development is most advanced.
The combination of sorafenib and gemcitabine is well tolerated, but appears to be insufficient for improving the results in terms of efficacy. Both drugs can act as radiosensitising agents, so their combination with radiation therapy appears to be a more appropriate approach for improving previous results.
The initial purpose of this study is to evaluate the optimum dose of sorafenib for the combination with gemcitabine and radiation therapy in patients with unresectable disease for, in a subsequent stage, establish the efficacy of this combination in patients with locally advanced disease, the primary endpoint being the assessment of progression free rate at 6 months.

4.2 Study objectives

PHASE I

Primary objective

To assess the safety profile and establish the maximum tolerated dose (MTD) / recommended dose (RD) of sorafenib in combination with gemcitabine and radiation therapy concomitantly.

PHASE II

Primary objective

To establish the antitumoural activity profile assessed as the percentage of progression-free rate (PFR) at 6 months, of the combination of sorafenib, gemcitabine and radiation therapy concomitantly in patients with locally advanced adenocarcinoma of the pancreas not receiving previous treatment.

Secondary objectives

- Percent response (PR/CR) by RECIST criteria.
- Overall survival.
- Toxicity occurring using the National Cancer Institute toxicity criteria (NCI CTC) version 3.0.
- Percentage of patients susceptible of surgery (R0) after treatment.

5.- CLINICAL TRIAL TYPE AND DESIGN

A phase I-II multicenter, non-randomised, open, two-phase study; a first phase to establish the optimum dose of the combination of sorafenib, gemcitabine and radiation therapy concomitantly the treatment of patients with locally advanced adenocarcinoma of the pancreas or metastatic disease not previously treated, and a second phase to establish the efficacy of this combination in percentage of progression-free rate at 6 months.

5.1 Design of the Phase I study

5.1.1 Administration of the study drugs

The patients included in this phase will receive the following combination at each dosage level investigated **during five consecutive weeks:**

Level 1:

- Sorafenib 200 mg daily administered po continuously.
- Gemcitabine 300 mg/m2/week in 30-minute iv infusion on Day +1 of each week.
- Radiation therapy on Day +1 to Day +5 of each week 1.8 Gy daily (1.8 Gy x 5 fractions x 5 weeks = 45Gy).

Level 2:

- Sorafenib 200 mg bid administered po continuously.
- Gemcitabine 300 mg/m2/week in 30-minute iv infusion on Day +1 of each week.
- Radiation therapy on Day +1 to Day +5 of each week 1.8 Gy daily (1.8 Gy x 5 fractions x 5 weeks = 45Gy).

Level 3:

- Sorafenib 400 mg bid administered po continuously.
- Gemcitabine 300 mg/m2/week in 30-minute iv infusion on Day +1 of each week.
- Radiation therapy on Day +1 to Day +5 of each week 1.8 Gy daily (1.8 Gy x 5 fractions x 5 weeks = 45Gy).

Table 3. Chart of phase I treatment.

Treatment duration	Weeks	Gemcitabine	Sorafenib	Radiation therapy
5 weeks receiving gemcitabine+sorafenib+radiation therapy	Week 1	Day 1	Daily	Days 1, 2, 3, 4 and 5
	Week 2	Day 1	Daily	Days 1, 2, 3, 4 and 5
	Week 3	Day 1	Daily	Days 1, 2, 3, 4 and 5
	Week 4	Day 1	Daily	Days 1, 2, 3, 4 and 5
	Week 5	Day 1	Daily	Days 1, 2, 3, 4 and 5

A maximum of 5 doses of gemcitabine will be administered. In case radiation therapy is delayed, the scheduled dose of gemcitabine will be continued for a maximum of 5 doses, continuing with sorafenib. Sorafenib will be discontinued when radiation therapy is delayed for over 3 days, and will be reinstituted when radiation therapy is restarted.
Treatment with sorafenib will be maintained until any of the criteria described in Section 6.3 of this protocol *"End of the study treatment"* is met.

The dose changes based on toxicity are described in Section 7. Toxicity will be evaluated with reference to NCI-CTCAE v. 3.0.
Adverse event (AE) and Serious Adverse Event (SAE) reporting is described in Section 9.

5.1.2 Definition of MTD, RD, and DLT

Definition of maximum tolerated dose (MTD)

The MTD is defined as the highest dose level that can be administered in a cohort of 6 patients where a maximum of 2 patients have experienced DLT.

Definition of recommended dose (RD)

The MTD will be considered as the recommended dose.

Definition of dose-limiting toxicity (DLT)

Limiting toxicity will be considered as grade 3-4 non-haematological or grade 4 haematological toxicity involving treatment discontinuation with radiation therapy for a period exceeding 2 weeks or any treatment-related death.

Table 4.Haematological toxicities for non-administration of gemcitabine, sorafenib or radiation therapy.

Haematological toxicities
Neutrophils <0.5 x 10^9/L
Febrile neutropenia:<br>• Neutrophils <0.5 x 10^9/L, and<br>• Fever ≥ 38.5ºC
Platelets <25 x 10^9/L

Table 5. Non-haematological toxicities for DLT.

**Non-haematological toxicities**
Related grade 3-4 non-haematological toxicities (occurring after optimum support therapy, if applicable) excluding:<br>• Nausea and vomiting not refractory to antiemetic treatment<br>• Hyperglycaemia<br>• Hypoglycaemia<br>• Deep venous thrombosis<br>• Hyperbilirubinemia secondary to malfunction of intraluminal stent
Symptomatic hypertension
Related grade 3 skin toxicity (rash) not controlled with general support measures

5.1.3 Dose escalation

The planned duration of combination therapy is five weeks. No dose escalations are permitted in the same patient.
Each cohort will include three patients.
For dose escalation, toxicity will be considered. Escalation of the dosage level will be performed in each cohort according to the optimum regimen shown in the table below. Before dose escalation, the patients of the cohort immediately below must have completed the treatment and the observation period.

Table 6. Dose escalation

No. of patients with DLT at a given dose level	Decision of dose escalation
0 of 3 patients	Escalate dose level
1 of 3 patients	The cohort will be extended including 3 additional patients.<br>• If 0 or 1 of the 3 patients in the extended cohort show DLT, it is escalated to the next dose level.<br>• If 2 or more of the 3 patients of the extended cohort show DLT, escalation to the next dose level will be stopped and the lower dose level is considered as DLT and RD.
≥ 2 patients of 3 patients	Escalation will be stopped at the next dose level.<br>The lower dose level is considered as MTD and therefore RD.<br>3 additional patients will be included in the lower dose level if only 3 patients were included in this level.

If the recommended dose of sorafenib is below 400 mg every 12 hours, the sponsor will re-assess the suitability of continuing with Phase II of the study at lower doses.

5.2 Design of the Phase II study

Phase II is a multicenter, non-randomised, open clinical trial on the combination of sorafenib, gemcitabine and radiation therapy concomitantly administered at the recommended doses in Phase I in patients with locally advanced adenocarcinoma of the pancreas not previously receiving treatment for the purpose of evaluating the efficacy considered as percentage of progression-free rate at 6 months.
The treatment administration is divided into three stages: Induction, combination therapy and maintenance. A total of 39 patients will receive treatment to progression or unacceptable toxicity.

The three treatment stages are:

Induction therapy:

The patients will receive the combination of gemcitabine and sorafenib **for 8 weeks :**

- Sorafenib at the RD established in the phase I study with continuous daily administration
- Gemcitabine 1000 mg/m2/week in 30-minute iv infusion for three consecutive weeks followed by a rest week for two cycles (one cycle= 4 weeks).

At the end of the induction treatment, a CT will be performed to evaluate the disease progression. Patients with tumoural regression will discontinue the treatment and will leave the study.
In the absence of tumoural progression, the patient will continue treatment in the next steps.

Combination therapy:

The patients will receive the combination of gemcitabine, sorafenib and radiation therapy **for 5 consecutive weeks :**

- Sorafenib at the RD established in the phase I study with continuous daily administration
- Gemcitabine 300 mg/m2/week in 30-minute iv infusion on Day +1 of each week.
- Radiation therapy on Day +1 to Day +5 of each week 1.8 Gy daily (1.8 Gy x 5 fractions x 5 weeks = 45Gy).

Maintenance therapy:

The patients will receive maintenance sorafenib until the end of the clinical trial or tumoural progression:

- Sorafenib 400 mg bid administered po until disease progression.

Table 7. Chart of phase II treatment.

Treatment duration	Weeks	Gemcitabine	Sorafenib	Radiation therapy
**Induction**<br>Treatment with gemcitabine (2 months) + sorafenib	Week 1	Day 1	Daily	
	Week 2	Day 1	Daily	
	Week 3	Day 1	Daily	
	Week 4	Rest	Daily	
	Week 5	Day 1	Daily	
	Week 6	Day 1	Daily	
	Week 7	Day 1	Daily	
	Week 8	Rest	Daily	
**Combination therapy**<br><br>5 weeks with gemcitabine + sorafenib (Phase I RD) + radiation therapy	Week 9	Day 1	Daily	Days 1, 2, 3, 4 and 5
	Week 10	Day 1	Daily	Days 1, 2, 3, 4 and 5
	Week 11	Day 1	Daily	Days 1, 2, 3, 4 and 5
	Week 12	Day 1	Daily	Days 1, 2, 3, 4 and 5
	Week 13	Day 1	Daily	Days 1, 2, 3, 4 and 5
**Maintenance**<br>Treatment with sorafenib 400 mg bid	Week 14		Daily	
	Week 15		Daily	
	Week 16		Daily	
	Week 17		Daily	
	Week 18		Daily	
	Week 19		Daily	
	Week 20		Daily	
	Week 21		Daily	
	Week 22		Daily	
	…		Daily	

6.- *SUBJECT SCREENING*

6.1 Inclusion criteria

To be included in the study, the patients must meet all inclusion criteria

and no exclusion criteria as indicated below:

1. Patients with histological or cytological diagnosis of adenocarcinoma of the pancreas with the following exceptions:
   A.- In Phase I of the study patients with adenocarcinoma of the pancreas will be included:
      I. Locally advanced inoperable according to the radiological criteria defined in Annex IX or intraoperative.[1]
      II. Patients with metastatic disease that, in the investigator's opinion, can benefit from treatment with local radiation therapy.
      III. Patients with local relapse who meet the criteria of unresectability and not receiving adjuvant treatment.
   B.- In phase II of the study only patients with locally advanced disease will be included (inoperable according to the radiological criteria defined in Annex IX or intraoperative1).
   C.- They should have received no previous chemotherapy, radiation therapy for the disease.

2. Presence of **measurable** disease defined by RECIST criteria. The assessment of the disease will be performed considering the following conditions (Annex IX details the radiology technique):
   - Multidetector CT (preferable) or spiral CT
   - Obtaining images with collimation and increase of 5 mm or less.
   - It will be performed at baseline conditions without the patient undergoing any surgical technique in the past 30 days and in the absence of bile prostheses.
   - Dynamic study in pancreatographic and portal venous phases.

[1] An intraoperative inoperability criterion is defined as the evidence to the surgeon during the procedure of peritoneal carcinomatosis and/or vascular or adjacent organ involvement not shown in imaging tests.

3. Patient (men and women) aged 18 years or older.

4. ECOG ≤ 1 in phase I and II.

5. Patients with an appropriate haematological, liver and renal function defined as:
    a. Neutrophils ≥ 1.5 x 109/L
    b. Platelets ≥100 x 10^9/L
    c. Haemoglobin ≥ 9 g/dL
    d. Total bilirubin < 1.5 times the upper normal limit.
    e. ALT and AST ≤ 2.5 times the upper normal limit (≤ 5 times the upper normal limit in case of liver involvement by the tumour)
    f. Serum creatinine ≤ 1.5 times the upper normal limit.
    g. INR ≤ 1.5 and APTT within normal ranges.
    h. Patients with creatinine clearance ≥ 45mL/min.
    i. Prothrombin time (PT) or INR and cephalin time (TTP) ≤1.5 times the upper normal limit.
6. Potentially fertile patients from both sexes must use appropriate contraceptive methods (barrier methods or other birth control methods) prior to entering the study and while participating in it. After withdrawing the treatment with BAY 43-9006, men should use contraceptive methods in the subsequent 6 months.
7. Patients giving their written informed consent before performing any specific study procedure.

6.2 Exclusion criteria

1. Patients diagnosed of adenocarcinoma of the pancreas receiving previous treatment for their disease.
2. With regard to radiation therapy, the patients must not show PTV (Blank planning volume) >500 cm^3
3. Patients allergic to iodinated contrasts or renal failure preventing the performance of the study and follow-up by radiological tests.
4. Pregnant or breast-feeding patients. Women with child-bearing potential will not have a positive pregnancy test, performed in the 7 days prior to the start of treatment.

5. Abuse of substances, clinical, psychological or social conditions that can interfere with the validity of the informed consent or protocol compliance.
6. Patients diagnosed of another malignancy except for cervical carcinoma in situ, treated basocellular carcinoma or superficial bladder tumours (Ta and TIS), or other malignant tumours receiving curing treatment >5 years before inclusion in the study.
7. Concomitant anti-tumoural treatment.
8. Patients receiving any experimental drug during the 30 years prior to inclusion in the study.
9. Patients on anticoagulation therapy with vitamin K antagonists (warfarin, acenocoumarol), with heparins or heparinoid derivatives. Low doses of warfarin are permitted if INR < 1.5. Low doses of aspirin are permitted.
10. Patients with any medical condition that can interfere with safety during participation in the study.
11. Significant weight loss (≥ 10% of body weight) in the 6 weeks prior to inclusion in the study.
12. Major surgery in the previous 3 weeks or biopsy by laparoscopic surgery or significant traumatic injury in the 2 weeks prior to the start of the study drugs. In patients with a history of surgery where the established time period has been met and that can be eligible for the study, the investigators should document the adequate surgical wound healing before starting the study therapy.
13. Patients with any contraindication or suspect allergy to the investigational products (sorafenib and gemcitabine).
14. Patients with a history or evidence of bleeding diathesis and/or coagulation disease.
15. Thromboembolic disease events, including stroke (with TIA) occurring in the past 6 months.
16. Uncontrolled hypertension defined as systolic blood pressure > 150 mmHg or diastolic blood pressure > 90 mmHg, despite appropriate medical therapy.
17. Patients with a history of cardiac arrhythmia requiring therapy with antiarrhythmics, unstable angina or new anginal episode starting in the past three months, acute myocardial infarction in the previous six months or congestive heart failure > NYHA class II.
18. Patients with class C liver disease in Child-Pugh classification.
19. Patients with (estimated creatinine clearance < 45 ml/min) or requiring dialysis.

20. Patients with active clinically serious bacterial or fungal infectious diseases (≥ grade 2 of NCI CTC, Version 3).
21. Patients with active wounds, ulcers or recent bone fractures.
22. Patients on concomitant treatment with ketoconazole, itraconazole, ritonavir, rifampicin, or St. John's wart (Hypericum perforatum).
23. Patients with known brain metastasis. In patients with neurological symptoms, a CT or MRI should be performed to rule out brain metastasis.
24. Any unstable condition or any that could endanger the safety of the patient and/or compliance.

6.3 End of the study treatment

The patients will end the study treatment for any of the following reasons:

1. Removal of consent by the patient or legal representative.
2. Evidence of disease progression.
3. Development of unacceptable toxicity in the investigator's opinion. The section on dose changes specifies the management of toxicities and recommendations for discontinuing the study.
4. If switching treatment is considered to be of interest for the patient, according to the investigator's criterion.
5. If the patient shows a beta-HCG compatible with pregnancy, reporting pregnancy as a serious adverse event.
6. Use of illegal drugs or other substances by the patient that, in the investigator's opinion, can reasonably contribute to toxicity or somehow interfere with the results.

After disease progression, the patients will be contacted regularly (every 3 months) and followed until death or study closure, to note the date of death (if this has not occurred yet). The subsequent treatments received will be entered in the CRF.

In case any protocol violation occurs, the sponsor should be asked to establish if the treatment must be discontinued. In case the treatment is discontinued for any reason other than disease progression,

a follow-up of the patient will be made as described in the procedures for follow-up until progression (i.e., with CT every 2 months).

If death occurs during the period of inclusion in the study or in the 30 days following the end of treatment, the responsible investigator will inform the principal investigator and the sponsor by sending the relevant SAE (serious adverse event) in the next 24 hours.

6.4 When and how the study subjects will be removed

In all cases of removal of a patient from the study, this should be notified to the monitor, completing the relevant section of the case report form (CRF).

Criteria for removal from the study

The only criteria for removal from the study are as follows:

1. Removal of informed consent.
2. Death of the patient.
3. Lost to follow-up.

The investigator will make every effort to keep all patients in the study unless it considered most appropriate for the patient to discontinue participation.

6.5 Sample size

Phase I:

A standard dose escalation study will be used, with cohorts of 3-6 patients. At least 3 patients will be treated in each dose level to evaluate toxicity. For inclusion of the third patient in a given dose level, the first 2 patients must have receive treatment and being monitored for the 5 weeks of the cycle to establish if DLT has occurred and if the third patient must be included in this level or not.

If 1 of 3 patients show DLT, at least 3 additional patients will be included in this dose level. The DLT is defined as the dose level where ≤ 2 patient of cohort of 6 experience DLT and the recommended dose (RD) will be the DLT.

Phase II:

The percentage of patients free from progression at 6 months is the primary endpoint of Phase II of the study. Considering that a percentage of PFR at 6 months $\leq 30\%$ as the definition of lack of efficacy of the treatment and a percentage of PFR $\geq 50\%$ as the definition of activity of treatment, with an alpha error of 0.05 and a beta error of 0.80 39 patients to demonstrate that the PFR is 50% or higher. If at least 17 patients or less are free from progression at 6 months, it can be concluded that the combination is active enough for subsequent development.
The percentage of progression-free rate will be established with the appropriate confidence interval. In addition, the Kaplan-Meier curve for progression-free survival will be obtained, calculating the median with the confidence intervals.

6.6 Estimated duration of recruitment period

Phase I.

A duration according to the times established for patient inclusion in each cohort until the recommended dose is found is planned, though a duration of 12 months is estimated.

Phase II

An inclusion period of 12 months is planned. However, entry rate will depend on the recruitment capacity at each of the centres participating in the study.

6.7 Patient registration system

After verifying that the patient meets all inclusion and no exclusion criteria, the log form will b sent to Pivotal by fax. The confirmation of patient registration and registry number will be sent by fax to the investigator.

7.- DESCRIPTION OF TREATMENT

7.1 Study drug

Phase I.

The patients registered will receive the following treatment by dose levels:

Table 8. Dose escalation chart

Dose escalation chart			
**Dose Level**	**Dose**		
	**BAY 43-9006 (Sorafenib)**	**Gemcitabine**	**Concomitant radiation therapy**
**Level 1**	200 mg/ every 24h	300 mg/m2	1.8 Gy fractionated in 5 doses/week for five weeks = 45 Gy
**Level 2**	200 mg every 12 hours		
**Level 3**	400 mg every 12 hours		

The duration of treatment is **five weeks**.

Sorafenib: 200-400 mg once or twice a day by oral route administered continuously. Film-coated tablets, dose 200 mg/tablet. It is recommended to administer the tablets fasting or with a mild moderate or low on fat.

Gemcitabine: 300 mg/m2/week in intravenous infusion in 100 ml of 0.9% saline for 30 minutes on Day +1.
A maximum of 5 doses of gemcitabine will be administered. In case radiation therapy is delayed, the planned dose of gemcitabine will be continued to a maximum of 5 doses, continuing with sorafenib, provided there is not toxicity that prevents treatment administration.
Sorafenib will be discontinued when radiation therapy is delayed for over 3 days, and will be reinstituted when radiation therapy is restarted.
Treatment with sorafenib will be maintained until any of the criteria described in Section 6.3 of this protocol *"End of the study treatment"* is met.

Concomitant radiation therapy: Total dose of 45 Gy (1.8 Gy/ fraction x 5 fractions x 5 weeks). Radiation therapy will be administered with linear accelerator without boost. The irradiated area will include the tumoural area plus a maximum margin of 2 cm, and the volume to be treated (PTV) ≤ 500 cm^3.

The definition of treatment volume will be performed by computerised tomography (CT), following the recommendations of the International Commission on Radiation Units and Measurements 50 Report. The tumoural volume (GTV) will be measured section by section in the CT planning by the software of the three-dimension treatment planner. The planned tumoural volume (PTV) will include the GTV with a lateral margin of 10 mm, anteroposterior of 10 mm and cranio-caudal of 20 mm. The risk organs (RO) to be considered will be the kidneys, the spinal cord, and the liver. All RO should be outlined for the purpose of generating dose-volume histograms and the maximum doses. The treatment will be performed by linear accelerator and with an energy of at least 6MV with isocentric technique. Customised protection blocks or multifoil system will be used for minimising the radiation dose in RO. For the purpose of preventing renal toxicity, it is recommended that a maximum of 50% of both kidneys do not exceed the dose of 20 Gy. It is recommended to limit the dose in the liver to 30 Gy for 50% of the liver volume. It is recommended to limit the dose in the spinal cord to 40 Gy.
In case the recommended dose of sorafenib is below 400 mg every 12 hours, the sponsor will re-assess the suitability of continuing with Phase II of the study at lower doses.

Phase II

The treatment is divided into three stages:

Induction therapy:

The patients will receive the combination of gemcitabine and sorafenib **for 8 weeks :**

- Sorafenib at the RD established in the phase I study with continuous daily administration
- Gemcitabine 1000 mg/m2/week in 30-minute iv infusion for three consecutive weeks followed by a rest week for two cycles (one cycle= 4 weeks).

At the end of the induction treatment, a CT will be performed to evaluate the disease progression.
Patients with tumoural regression will discontinue the treatment and will leave the study.
In the absence of tumoural progression, the patient will continue treatment in the next steps.

Combination therapy:

The patients will receive the combination of gemcitabine, sorafenib and radiation therapy **for 5 consecutive weeks :**

- Sorafenib at the RD established in the phase I study with continuous daily administration

- Gemcitabine 300 mg/m2/week in 30-minute iv infusion on Day +1 of each week.
- Radiation therapy on Day +1 to Day +5 of each week 1.8 Gy daily (1.8 Gy x 5 fractions x 5 weeks = 45Gy).

Maintenance therapy:

The patients will receive maintenance sorafenib until tumoural progression:

- Sorafenib 400 mg bid administered by oral route until disease progression.

A CT will be performed every 2 months until disease progression is documented. In case of withdrawal from the study for any reason other that disease progression (see end of study criteria), the follow-up should be performed every 2 months as established in the study until documenting disease progression. In case of no radiological documentation of disease progression, the date of the last CT performed with no progression documentation will be the date of disease progression. In case the patient has only completed the baseline CT the date of disease progression will be Day +1 after the date of inclusion of the patient in the study. Patients with tumoural regression will discontinue the treatment and will leave the study.

7.2 Dose Modifications

The doses will be modified in case of severe haematological and/or non-haematological toxic effects.
The doses will be reduced following these levels**:**

Table 9. Dose changes

**Drugs:**	**Gemcitabine**	**Sorafenib**
**Starting dose**	To be established in Phase I	To be established in Phase I administered every 12 hours
**Level -1**	Reduction of 25% of the starting dose	
**Level -2**	Reduction of 50%	

With regard to dose adjustments, the following general recommendations apply.

During chemotherapy-radiation therapy:

- In case of grade 4 haematological or grade 3-4 non-haematological toxicity, the treatment with gemcitabine, sorafenib and radiation therapy will be stopped until the toxicity subsides to grade 1. In case of haematological recovery (>1000 N and >75,000 platelets) or grade 1 non-haematological, treatment with sorafenib can be restarted at the starting dose, gemcitabine with a dose reduction of 75% of the starting dose of gemcitabine and radiation therapy. In case of grade 3 thrombocytopenia (25,000-50,000) chemotherapy will be discontinued but treatment with sorafenib and radiation therapy. In this case a blood count every 48 hours is recommended to establish the outcome of platelets.

  Limiting toxicity will be considered as grade 3-4 non-haematological or grade 4 haematological toxicity involving treatment discontinuation with radiation therapy for a period exceeding 2 weeks or any treatment-related death. A maximum of 5 doses of gemcitabine will be administered. Whenever possible and if the abovementioned dose changes allow fir it, gemcitabine will be administered as planned in case of discontinuation of radiation therapy.

During the induction treatment:

- If in one cycle any of the doses of gemcitabine of Day 8 or Day 15 cannot be administered, these doses will not be recovered and the treatment will be continued on Day 28 as soon as baseline levels or grade ≤ 1 have been recovered.
- If grade 4 haematological toxicity occurs, the dose of gemcitabine will be discontinued, when it has been recovered to grade ≤ 1 the treatment may be re-started and the dose of gemcitabine will be reduced one dose level. In case the dose should be reduced more than one level, the treatment with gemcitabine will be discontinued and the patient may continue on sorafenib or end the study treatment following investigator’s judgement.
- For the administration of chemotherapy at full doses, absolute neutrophil count (ANC) should be ≥ 1.0 x 109/L cells and platelet count should be ≥75 x 109/L before administration of chemotherapy.
- In case of 500-1000 N and 50-75,000 platelets, gemcitabine can be administered at 75% of the dose. If <500 N or <50,000 platelets, treatment with gemcitabine will not be administered.

- If sorafenib should be discontinued for unmanageable toxic effects with dose reduction, treatment with gemcitabine will be continued. If it should be discontinued for over four weeks, the patient will leave the study.
- If a patient experiences several toxic effects and the recommendations to be followed are conflicting, consider the most conservative dose adjustment of those recommended (appropriate dose reduction for the most severe toxicity). Note that once the dose has been reduced for toxicity, it should not be increased again.
- If the patient requires a major surgical procedure or experiences a bleeding ulcer during the study, the treatment with sorafenib will be discontinued and will be re-started when the appropriate wound healing occurs (approximately about 4 weeks).

The toxic effects will be classified, whenever possible, according to the NCI CTCAE v3.0.
The reasons for dose changes or treatment delay as well as the support measures adopted and the outcome will be entered in the clinical history and the CRF.

7.2.1 Non-haematological toxicity

7.2.2.1 Hand-foot syndrome related to sorafenib

Classification of the hand-foot syndrome

**Grade 1**	Numbness, dysesthesia/paresthesia, tingling, painless swelling or erythema of the hands and/or feet and/or disorders not interfering with activities of daily life.
**Grade 2**	One or more of the following symptoms: painful erythema and swelling of the hands and/or feet and/or disorders interfering with the daily activities of the patient.
**Grade 3**	One or more of the following symptoms: wet flaking, ulcers, blisters or severe pain in the hands and/or feet and/or severe discomfort that prevent the patient from working or fulfilling activities of daily life.

<u>Grade ≥ 2 hand-foot syndrome:</u> If grade ≥ 2 HFS occurs, one dose level of sorafenib will be reduced.

7.2.2.2 Hypertension

When treatment with sorafenib is started, blood pressure should be monitored at least weekly during the first 6 weeks, then it will be monitored according to the standard medical practice.

- If asymptomatic grade 1 or grade 2 hypertension occurs, with diastolic blood pressure < 110 mmHg occurs, antihypertensive therapy should be started, continuing with sorafenib.
- If the patient experiences persistent grade 2 symptomatic hypertension or diastolic pressure =110 mmHg or grade 3 hypertension, the sorafenib will be discontinued until the symptoms subside and diastolic blood pressure is = 100 mmHg. In addition, antihypertensive therapy will be started and when treatment is re-started the starting dose of sorafenib will be maintained.
- If grade 4 hypertension occurs, the study treatment will be discontinued according to the investigator's judgement.

7.2.2.3 Skin rash related to sorafenib

Treatment with sorafenib can be associated with rash in the face and body appearing in the first 6 weeks of treatment. There are three types of skin rashes:

- Erythematous and flaky skin rash in the face and eyebrows and hairline.
- Maculopapullar skin rash in the body.
- Erythematous skin rash in the body, with small pustules.

They usually subside over time and the management is conservative with topical use of moisturising creams. As a general measure, it is not mandatory to discontinue treatment. If the skin injury worsens, becomes blistery or conjunctival involvement is suspected, the patient should be referred to a dermatologist and it will be considered as grade 4 toxicity.

7.2.2.4 Other non-haematological toxicities not specified above

Grade ≤2 toxicity	Treatment with sorafenib and gemcitabine will be maintained during the cycle, except for the case of skin toxicity where treatment with sorafenib should be discontinued.
Grade 3-4 toxicity, first episode	Discontinuation of treatment with gemcitabine or sorafenib based on the type of toxicity until the toxic effect subsides to grade ≤ 1.<br>Once recovered to ≤ grade 1, it may be retreated with the lower dose level. (Sorafenib at the starting dose)
Grade 3-4 toxicity, second episode	If subsequently the same toxic effect ≥ grade 3 occurs, the same procedure will be followed, with a new reduction of the dose of gemcitabine, until recovery of grade ≤ 1
Grade 3-4 toxicity, third episode	If despite this toxicity ≥grade 3 recurs, gemcitabine will be discontinued.

7.3 Treatments permitted and prohibited before and/or during the study

7.3.1 Permitted treatments

- Patients may receive palliative and supportive care for any underlying illness.
- Patients receiving bisphosphonates as prophylaxis for bone metastasis can continue in the study while they receive treatment.
- The use of G-CSF in the first treatment cycle is not permitted G-CSF and other haematopoietic growth factors can be used for the management of acute toxicity, including febrile neutropenia when clinically indicated or in the investigator's opinion; however, their use must not replace a dose reduction for toxicity in case it is required.
- The use of supportive care, that can include analgaesics and nutritional support, is permitted.

- Patients with discomfort due to hand-foot syndrome may receive topical emollients, low-potency topical steroids, or urea-based creams.

7.3.2 Prohibited treatments

The use of drugs metabolites by the cytochrome CYP-3A4 enzyme or inducing or inhibiting CYP-3A4 must be avoided. In the event they are required, they should be used with caution and be closely monitored:

SUBSTRATES	INHIBITORS	INDUCERS
Amiodarone	Chlarithromycin	Barbiturates
Alprazolam	Cyclosporine	Carbazepine
Buspirone	Delaviridine	Griseofulvin
Cisapride	Erythromycin	Nevirapin
Cyclosporine	Fluconazole	Phenytoin
Diltiazem	Indinavir	Rifampin
Erythromycin	itraconazole	St. John’s wart
Phelodìpine	Ketoconazole	
Indinavir	Miconazole	
Lovastatin	TAO	
Midazolam	Nefazodone	
NifedipinePimozide	Ritonavir	
PioglitazoneQuinidinae	Verapamil	
Ritonavir	Grapefruit juice	
Sertralin		
Sildenafil		
Simvastatin		
TAO		
R-Warfarin		
Tacrolimus		
Triazolam		
Verapamil		
Zolpidem		

The patients taking drugs with a narrow therapeutic margin, including anticoagulants, phenytoin, quinidine, carbamazepine, phenobarbital, cyclosporine and digoxin, must be monitored

proactively.
The following treatments are not permitted during the study either:

- Megestrol acetate and medroxyprogesterone.
- Bone marrow transplant or rescue with stem cells
- Bevacizumab and other drugs (under study or approved) with VEGF as target.
- Study treatment or other anti-cancer treatment approved other than that specified in the patient screening criteria (immune therapy and/or chemotherapy, etc.), except for bisphosphonates.

7.4 Procedures for monitoring treatment compliance by the subject

To evaluate appropriate compliance, the drug will be counted, entering in the CRF the drug dispensed and collected.

8.- ASSESSMENT CRITERIA AND STUDY CONDUCT

8.1 Evaluation criteria: Definition of populations

The patients will be grouped in three categories:

1. The population by intention-to-treat (ITT) will include all patients registered and receiving one dose of the drug.
2. The population per protocol (PP) will include the set of patients from the ITT population with no major protocol deviations and receiving one dose of drug.
3. Response evaluable population: to be included in this population, they must meet the following characteristics:
    a. Be eligible, having a measurable disease.
    b. Completing the evaluation of all baseline injuries at least once with the same techniques used at baseline.
    c. No major protocol violations (such as concomitant anti-cancer treatment).

4. Patients evaluable for toxicity: all patients will be evaluable for toxicity since they have received a first administration of the study drugs.

8.2 Efficacy assessment

These assessments will be made in both the population by intention-to-treat (ITT) and the population per protocol.

Progression free rate at 6 months

Percentage of disease progression (radiological) free patients defined according to the study.

Progression-free survival

Time from inclusion in the study to disease progression.

Objective response (OR)

The objective response rate is defined as the percentage of patients reaching a complete (CR) or partial response (PR): the response will be assessed following RECIST criteria.

Overall survival

Overall survival will be established from the date of inclusion in the study to the date of death for any reason.
Patients lost to follow-up will be censored at the date of last follow-up.

Percentage of surgical resection
The patients who after the study treatment were susceptible of surgical resection. Patients with surgery R0 (gross resection with negative pathological margins) will be distinguished from those with R1 (gross resection with positive pathological margins) and with surgery R2 (gross residual tumour).

8.3 Safety assessment

Each patient will be monitored regularly for the detection of possible adverse events. For assessment, the NCI-CTCAE v3.0. will be followed. The toxic effects that cannot be classified according to the toxicity criteria of the above system will be rated as follows (MedDra classification):

- Mild (asymptomatic).
- Moderate (symptomatic, but does not significantly interfere with function).

- Severe (causes a significant interference with function.)
- Life-threatening.

8.4 Timetable of assessments

8.4.1 Planned assessments on inclusion and during treatment

All patients included must be assessed according to the timetable below.
The following examinations will be performed at the time points established in the enclosed table:

- Clinical history and physical examination including weight, performance status and blood pressure. In the case of the first cycle, these data should be collected in the seven days prior to the start of treatment. In the part of Phase I and during the concomitant phase of Phase II, the visits will be weekly and every four weeks during the part of induction and maintenance of Phase II. During the treatment, BP will be measured weekly during the first 6 weeks.
- Baseline and toxicity symptoms evaluated according to the NCI-CTCAE v3.0 toxicity criteria (if the NCI classification is not applicable, the MedDra classification will be used).
- Haematology with count of the three series (WBC with neutrophils, haemoglobin, platelets). In the case of the first cycle, these data should be collected in the seven days prior to the start of treatment. In Phase I, in the induction phase (weekly except for the third week) and concomitantly of Phase Ii they will be performed weekly. During the maintenance phase, the following will be performed every four weeks.
- Biochemistry: alkaline phosphatase, ASAT (SGOT), ALAT (SGPT), bilirubin, serum creatinine, creatinine clearance (if indicated), amylase and lipase. In the case of the first cycle, these data should be collected in the seven days prior to the start of treatment. In Phase I, in the induction phase (weekly except for the third week) and concomitantly of Phase Ii they will be performed weekly. During the maintenance phase, the following will be performed every four weeks.
- Prothrombin time and INR. These data should be collected in the 7 days prior to the start of treatment.
- Pregnancy test in women with child-bearing potential.
- ECG in the 2 weeks prior to the start of treatment.

- Imaging tests required for assessing the tumour: Spiral or multidetector CT or any other specific test. In the case of the first cycle, these data should be collected in the four weeks prior to the start of treatment. In Phase I and Phase II, the tumour will be assessed every 2 months until disease progression.
- Bone scintigram if indicated.
- Other investigations as clinically indicated, including ECG.

		**PHASE I**	**PHASE II**
**History and physical examination**[1]	Clinical history<br>Physical examination: weight and performance status and blood pressure.	In the seven days prior to the start of treatment in the first cycle. Weekly during the treatment.<br>Blood pressure will be measured weekly during the first 6 weeks.	In the seven days prior to the start of treatment in the first cycle.<br>During the induction and maintenance treatment it will be performed every four weeks. During concomitant treatment weekly.<br>Blood pressure will be measured weekly during the first 6 weeks then every 4 weeks before each cycle.
**Baseline and toxicity symptoms (adverse events)**	Evaluation of toxicity according to NCI criteria.	In the seven days prior to the start of treatment in the first cycle. Weekly during the treatment.	In the seven days prior to the start of treatment in the first cycle. During the induction and maintenance treatment it will be performed every four weeks. During concomitant treatment weekly.
**Haematology**[2]	Count of the three series and neutrophils	In the seven days prior to the start of treatment in the first cycle. Weekly during the treatment and every 2-3 days in case of count below 0.5 x 10^9 x L, grade 4 thrombopenia or febrile neutropenia, until recovery.	In the seven days prior to the start of treatment in the first cycle. During the treatment, they will be collected weekly during induction treatment except for the third week of treatment and during combination therapy.<br>During the maintenance phase, every four weeks. Every 2-3 days in case of count below 0.5 x 10^9 x L, grade 4 thrombopenia or febrile neutropenia, until recovery.
**ECG**		In the 14 days prior to the start of treatment.	In the 14 days prior to the start of treatment.
**Pregnancy test**		In the 7 days prior to the start of treatment.	In the 7 days prior to the start of treatment.
**Prothrombin time and INR**		In the 7 days prior to the start of treatment.	In the 7 days prior to the start of treatment.<br>Every 4 weeks before each cycle.
**Biochemistry**[2]	Alkaline phosphatase, ASAT (SGOT), ALAT (SGPT), bilirubin, serum creatinine, creatinine clearance (if indicated), amylase and lipase.	In the seven days prior to the start of treatment in the first cycle. Weekly during the treatment.	In the seven days prior to the start of treatment in the first cycle. During the treatment, they will be collected weekly during induction treatment except for the third week of treatment

		PHASE I	PHASE II
			and during combination therapy. During the maintenance phase, every four weeks.
**Imaging/measurement of tumour**[3]	Examinations required for tumour assessment. Spiral or multidetector CT. Echoendoscopy Using the same technique as in the baseline assessment. **The instructions detailed in Annex XI for performing the CT will be followed.** Bone scintigram if indicated.	In the four weeks prior to recording, and at Week 7 after the start of treatment (2 weeks after the end of treatment); then every 2 months until disease progression is documented. Tumour assessments must be maintained at the time points established from the recruitment of the patient.	In the four weeks prior to recruitment, and subsequently a CT will be performed at the end of the induction treatment. If the patient continues in the study a CT will be performed every 2 months until disease progression. Tumour assessments must be maintained at the time points established from the recruitment of the patient.
**Other investigations**	Others, as clinically indicated	As clinically indicated	As clinically indicated

1 The concomitant drugs will be noted at baseline in the relevant CRFs, including all drugs used one month before recruitment. For ALL the other visits, the concomitant drugs will be only noted ONLY if these are related to adverse events.

2 If possible, laboratory measurements will be performed at the same laboratory during the study. The laboratory values must be expressed with the normal ranges for each parameter.

3 To assure comparability, the baseline radiographic tests and those used to demonstrate the response must be performed using identical techniques (e.g., CT must be performed, provided the patient conditions allow for it, after bolus injection of intravenous contrast using a standard contrast volume, identical agent, and preferably the same scanner). The instructions detailed in Annex will be followed. **An attempt will be made to follow each lesion with the same instrumental examination from baseline to the follow-up.** The minimum interval to compare the response by two radiological tests must be four weeks.

8.4.2 End of Treatment Visit

This visit will be performed between 21 and 30 days after the end of treatment both with radiation therapy-chemotherapy and sorafenib (including patients not completing all cycles). The procedures will include: a physical examination, haematology and biochemistry tests, examinations to assess the response if the patient has not progressed previously, noting toxicity. If the patient has not progressed when discontinuing the study, tumour assessments every 2 months will be continued, until disease progression. All end of treatment assessments will be noted in the last cycle of the CRF.

8.4.3 Follow-up assessments

After disease progression, the patients will be followed according to clinical criterion until death, to document:

- Follow-up of side effects, if any, until resolution.
- Subsequent treatment.
- Survival.

IF THE PATIENT WAS REMOVED FROM THE STUDY BEFORE PROGRESSION, ALL CLINICAL AND RADIOLOGICAL ASSESSMENTS OF ALL LESIONS SHOULD BE PERFORMED EVERY 2 MONTHS UNTIL PROGRESSIVE DISEASE IS DOCUMENTED.

8.2.4.- Withdrawals

The reason for (e.g. consent withdrawal, adverse event, lost to follow-up, etc.) and date of withdrawal must be documented in the CRF of all patients discontinuing the study.
When a patient is withdrawn from the study, the investigator will make an attempt to complete all end of study procedures.

9.- SAFETY. REGISTRATION AND REPORTING PROCEDURE

9.1 Adverse event

An adverse event is any unfavourable medical event seen in a patient or investigational subject receiving a drug. The adverse event does not necessarily need to have a causal relationship with this treatment. Therefore, an adverse event is any disease, symptom or unfavourable unintended sign (including a laboratory abnormality) associated in time with the use of an investigational product, whether considered or not to be drug-related.
The adverse events associated with the use of a drug in humans, whether considered or not to be drug-related, comprise:

- An adverse event occurring during the use of a drug in the medical practice.

- An adverse event derived from overdosing, either accidental or intentional.
- An adverse event derived from drug abuse.
- An adverse event derived from drug withdrawal.
- When there is reasonable possibility that the adverse event occurs only due to the participation of the patient in the study (e.g., adverse event or serious adverse event caused by discontinuation of antihypertensives during wash-out), it should be also notified as an adverse event, even if not related to the investigational product.

The clinical manifestation of failure of the planned pharmacological action (progression) will not be recorded as adverse event if already noted in the data collected in the CRF. However, if the episode meets any of the criteria for “serious” adverse event, it should be registered and reported as such.

9.2 Serious Adverse Experience (SAE)

A serious adverse event (SAE) is defined as any unfavourable medical event that, at any dose:

- Results in death
- Is life-threatening.
- Requires or prolongs hospitalization
- Causes persistent or significant disability.
- Is a congenital abnormality or birth defect.
- Causes a significant medical event.

Is life-threatening: the term “is life-threatening” used in the definition of “serious” makes reference to an adverse event whereby the subject ran the risk of dying when it occurred. It does not refer to an adverse even that theoretically may have caused death if it had been more severe.

Hospitalisation: any adverse event leading to or prolonging hospitalisation will be considered as serious, EXCEPT for any of the following:

- The admission involves a hospital stay of less than 12 hours.

  or

- The admission was planned in advance (that is, surgery programmed before the start of the study).

  or

- The admission is not related to an adverse event (e.g., social hospitalisation to relieve the care provider).

However, it must be noted that the invasive treatment applied during any hospitalisation can meet the criteria of "medically important" and, as such, must be reported as a serious adverse event depending on the clinical judgement. Furthermore, when local regulatory authorities require specifically a more accurate definition, local regulations will prevail.

Disability means a substantial change in the ability of a subject to fulfil his activities of daily life.

Important medical event: an adverse event may be considered serious if it endangers the subject and requires an intervention to prevent other serious disease. The WHO publication "WHO Adverse Reaction Terminology – Critical Terms List" is used as guide to establish important medical events. The terms collected refer to or can indicate a serious morbid state.
These events require special attention as they can be associated with a serious disease and lead to more drastic measures than the reports made using other terms.
Disease progression or worsening of the existing cancer will be reported as a SAE regardless of meeting the criteria of "serious". The investigators should be reported in addition to the signs and symptoms occurring in the association with disease progression or worsening of the existing cancer.

9.3 Unexpected Adverse Event

An unexpected adverse event is any adverse drug reaction with a specificity or severity not consistent with those established in the current investigator's brochure (or in the package leaflet if the product is already marketed). Also, reports which add significant information on specificity or severity of a known, already documented adverse event constitute unexpected adverse events. For instance, a more specific or more severe event than described in the investigator's brochure must be considered to be "unexpected".

Some specific examples are:

a. Acute renal failure reported as adverse event followed by a new subsequent report of interstitial nephritis, and

b. Hepatitis with a first report of fulminant hepatitis.

9.4 Relationship of the adverse event with the investigational product

The assessment of the relationship of an adverse event with the administration of the study drug is a clinical decision based on all information available when completing the case report form.
A “negative” assessment means:

1. The existence of a clear alternative explanation, for instance, mechanical bleeding in the surgical area.

   or
2. Unlikeliness; for instance, the subject is knocked down by a car and there are no evidences that the drug caused the confusion that could lead to the accident; the appearance of cancer some days after the first drug administration.

A “positive” assessment indicates that there are reasonable suspects that the adverse event is associated with the use of the investigational drug.
The factors to be considered when assessing the relationship of the adverse event with the study drug are:

- Temporal sequence from study administration: the event must occur after the administration. The time from drug exposure to the event in the clinical setting of the event will be assessed.

- Recovery with discontinuation (withdrawal from exposure) and relapse with rechallenge (repetition of exposure): the response of the subject after drug discontinuation (withdrawal of exposure) or after drug rechallenge (repetition of exposure) will be considered considering the clinical outcome of the event involved.

- Underlying, concomitant, intercurrent diseases: any report will be assessed considering the natural history and outcome of the disease treated and of any other disease that can be suffered by the subject.

- Concomitant drugs or treatments: the other drugs taken by the subject or the treatment received should be evaluated to establish if any of them may have caused the event involved.

- Pharmacology and pharmacokinetics of the study drug: the pharmacokinetic properties (absorption, distribution, metabolism, and excretion) of the study drugs, in addition the pharmacodynamics of the given subject, will be considered.

9.5 Severity of the adverse event

- The severity of the adverse events will be rated according to the toxicity criteria of the National Cancer Institute (NCI CTC) version 3.0:

  - Grade 1: Mild adverse event
  - Grade 2: Moderate adverse event
  - Grade 3: Serious adverse event
  - Grade 4: Life-threatening or disabling adverse event.
  - Grade 5: Death related to the adverse event

9.6 Adverse event documentation

All adverse events occurring during the study all serious adverse events, regardless of their causal relationship with the study drug, occurring after the subject has signed the informed consent should be recorded in detail in the case report form of the subject.

The documentation must be supported by a note in the history of the subject. Any anomaly in a laboratory test considered clinically significant, for instance, leading the subject to withdraw from the study, requiring treatment or causing apparent clinical manifestations or that the investigator considers relevant, should be reported as adverse event. Every event will be described in detail together with the date of onset and end, severity, relationship with the investigational product, measures adopted, and outcome.

9.7 Serious adverse event/pregnancy reporting

Serious adverse events (SAE), including laboratory test abnormalities that meet the definition of serious, occurring after signing the informed consent and during the follow-up period should be reported immediately (within 24 hours since the investigator knows the event) to the sponsor, to the subject indicated in the study file. A serious adverse event form will be also completed within 24 hours after the investigator became aware of the event, and will be sent to PIVOTAL.
All serious adverse events will be monitored until they subside or become stable by sending updated reports to the designated individual.
The sponsor is responsible for reporting all SAEs or unexpected AEs that may be related to the investigational treatments occurring during the study to the Spanish Medicines Agency (SMA), the competent bodies of the Autonomous Communities where the trial is conducted, the Ethics Committees (EC), and investigators involved in the trial within the timelines established by the

applicable Spanish regulations.

Although they are not considered to be a serious adverse event, pregnancies occurring during a clinical investigation should be reported within the same timelines a serious adverse event. Pregnancies of both patients included in the trial and the partners of participating patients will be reported. The outcome of pregnancy will be closely monitored and any anomaly suffered by the mother or the child will be notified. All of this is also applicable if pregnancy occurs as a result of the sexual relations held by the father after the administration of the investigational product.

A report sheet sample is enclosed in an annex (ANNEX VIII. SAE report form)

10.- ETHICS

10.1 General considerations

The study will be performed following the guidelines specified in the Declaration of Helsinki, the Good Clinical Practice guidelines, ICH guidelines (International Conference on Harmonisation) and in compliance with current regulations.
The study will be performed in compliance with protocol requirements. Before the study start, the protocol and any relevant amendment, and the patient information sheet and informed consent will receive the approval/favourable opinion of the Clinical Research Ethics Committee (CREC) and the Spanish Agency for Medicinal Products and Medical Devices. For non-relevant amendments, the procedures established in the current law will be followed.

The informed consent from each subject should be obtained in writing and will be given freely before participation in the clinical trial.
The rights, safety and wellbeing of the study subjects should prevail over the interests of sciences and society.
The study staff involved in performing this study will be qualified for fulfilling the tasks assigned and any staff sanctioned or suspended for scientific fraud or clinical malpractice may not participate in it.

10.2 Clinical Research Ethics Committee (CREC)

Before starting the study, written approval or favourable opinion should be obtained from the CREC of this protocol, the patient information sheet, the informed consent form, and any document used for patient recruitment. The CREC will be also given the investigator's brochure and any update of the abovementioned documents.
The sponsor or if delegated the holder of authorisation of the investigational product should send to the CREC the reports, updates and any other information, including safety reports, protocol amendments, administrative letters according to the requirements from regulatory authorities or institution procedures.

10.3 Patient information and informed consent

The informed consent will be obtained considering the current law and the Declaration of Helsinki and its modifications.
Before including the patient in this trial, the investigator should duly inform the patient. The patient will give his consent after receiving all relevant information adapted to his level of understanding, and should be documented by a patient information sheet and the consent form. The consent should evidence the express agreement of the subject and may be withdrawn at any time without any damage to him/her.
The investigator should give the subject time to ask about the study details. Then the subject will sign and date the informed consent in person. The investigator should provide the subject with a copy of the consent form and the patient information sheet.

The informed consent and any other information provided to the subjects should be reviewed provided new relevant information is available on the voluntary participation of the subject and should receive the approval/favourable opinion from the CREC before implementation. The investigator or designee should fully inform the subject of all relevant study issues, and of any new information about the willingness of the subject to continue participating in the study. This report should be documented.
The subject participating in the clinical trial may withdraw the consent at any time, without specifying the cause and without this resulting in any responsibility or damage.

10.4 Confidentiality

The investigator will assure that all subjects involved will abide by the confidentiality of any information about the trial subjects.
All parties involved in a clinical trial will keep utmost confidentiality so that the personal or family privacy of the subjects participating in it is not broken. Furthermore, the appropriate measures should be adopted to prevent the access of non-authorised people to the trial data.
The handling of personal data on the subjects participating in the trial, particularly with regard to the consent, will comply with the provisions of Organic act on the protection of personal data, 15/1999 of 13 December and European Directive on Data Confidentiality. Your data will be object of a dissociated treatment, so that the information obtained cannot be associated with an identified or identifiable subject.
The data obtained in this study will be reviewed by a monitor designated by the sponsor and will be used only to draw scientific conclusions. The identity of the patients is confidential and will be only recognised by the investigator and his collaborators, auditors and monitors or agents authorised by the sponsor and inspectors from competent authorities.

11.- PRACTICAL CONSIDERATIONS

11.1 Responsibilities of the study participants

Rules for the patient

The patient should follow the instructions of the investigators and report any event to them.

The patient should be duly informed of the prohibitions or restrictions to be followed during the trial. Non-compliance with these recommendations will involve withdrawing from the study.
The patients may terminate their participation in the study at any time.

Rules for the research staff
The investigator undertakes to follow the rules established in the current law on clinical trials.

11.2 Monitoring, audit, and inspection

The study will be monitored by regular visits and telephone calls to the investigator. During visits to the centre, the monitor must review source patient records, drug supply logs, and document retention. The monitor must also evaluate study procedures and discuss any eventual problems with the investigator. During the course of the study, audit visits can be made to the participating centres. The investigator will allow direct access to source data/documents for the purposes of monitoring, audit, CREC review, and inspection by regulatory authorities.

11.3 Recording, handling and filing of data

The sponsor will provide the CRFs, that will include an original in white colour and several coloured copies. The forms should be completed in **LEGIBLE UPPERCASE**, using black ink. The trial monitor will verify the forms, comparing them to the original data recorded (and the worksheets, if applicable), before collection. The last copy will be kept in the investigator's files while the others will be returned to the sponsor. No CRF will be sent by mail to the sponsor without specific authorisation. CRFs and all the source data must be easily accessible for review during the programmed monitoring visits.

11.4 Files and reports

The investigator should prepare and keep the clinical histories, recording all examinations and relevant data for the investigation in each subject treated with the investigational drug. The data recorded in the CRF, obtained from the source documents, should be consistent with these source documents, or in case of discrepancies, these should be explained.

The confidentiality of the documents that could identify the subjects, respecting their privacy and confidentiality rules according to the applicable regulatory requirements, should be protected.
The investigator will keep a signature sheet to document all people authorised to enter data and/or amendments in the CRFs. An amendment must be made drawing a line on the wrong entry, and indicating the appropriate information
in the adjacent space. The amendment should include the initials of the individual entering it, the date of it, and the explanation (if appropriate). The original entry should not be masked.
The completed CRFs should be reviewed, signed and dated immediately by a qualified physician, that will be an investigator or a collaborator. The investigator should keep a copy of the CRFs including changes and amendments.

11.5 Retention of files

The sponsor is responsible for the file of the trial documentation. The investigator will keep the identification codes of patients for at least 15 years after completing or discontinuing the trial. The clinical histories of the patients and other original data will be kept for the maximum time period permitted by the hospital, institution or clinic where the study was performed.
The investigator should contact the sponsor before destroying any records associated with the study. The sponsor will inform the investigator when retention of the trial files is not longer necessary.
If the investigator withdraws from the study, the records should be transferred to another individual designated as mutually agreed. The sponsor will notify this transfer to the CREC and the Spanish Agency for Medicinal Products and Medical Devices.

11.6 Protocol amendment

Any protocol amendment should be documented in writing as an amendment. Amendments will be duly identified by their number in chronological order, dated and signed by the sponsor and investigator.
All protocol amendments will be reported to the CRECs involved in the trial, the AEMPS, and Autonomous Communities before they are implemented. For relevant amendments, approval from the involved CRECs and AEMPS is also required.

11.7 Use of information and publication

All study drug information provided by the sponsor in relation to this trial and not previously published is considered confidential information the property of the sponsor. Such information comprises the basic product information, the clinical protocol, any workbooks if applicable, CRFs, assessment methods, sponsor technical methods, and basic scientific data. This confidential information will be the property of the sponsor, must not be disclosed to third parties without prior written consent from the sponsor, and must only be used for the study purposes.
The information resulting of this clinical trial is also considered to be confidential and will be used by the sponsor in relation to the development of the study drug. Such information can be disclosed to the extent considered necessary by the sponsor.
In order to allow use of information derived from this study and to ensure compliance with the applicable regulations, the investigator is committed to provide the sponsor all results of the tests

and all data collected in this study. Except as required by the law, the information obtained during the study may be only provided to physicians and regulatory authorities by the sponsor, and may not be published or transferred, either complete or in part, to other parties, without previous written consent from the sponsor.
The publication of the data will be decided by the study sponsor after asking the investigators involved. The results cannot be published individually before the global publication of the study.
The publications will show all investigators including at least 2 of the planned patients. The coordinating investigator will be shown as the first author followed by the other investigators by order patient inclusion number.
The sponsor undertakes to publish the results, both favourable and unfavourable, of this trial.

11.8 Quality control and assurance

For the purpose of verifying protocol compliance, the agents assigned by the sponsor will be allowed for visiting the study centres regularly to verify data completeness, validate quality and accuracy of the study. The study documents will be reviewed at the centre comparing them to the source documents (for instance, clinical histories), will be compared with the investigator and the suitability of the facilities will be evaluated continuously.
The study may be assessed by in-house sponsor auditors or authorised agents and inspectors designated by regulatory authorities that may have access to the CRFs, source documents, and other study files. The audit reports will be kept confidential.

The investigator should immediately notify to the sponsor any inspection by regulatory authorities and provide a copy of the reports thereof.

12.- STATISTICAL ISSUES

12.1 Statistical methods

12.1.1 Efficacy population:

The patients will be grouped in three categories:

1.- The population by intention-to-treat (ITT) will include all patients recruited and receiving one dose of the drug.
2.- The population per protocol (PP) will include the set of patients from the ITT population with no major protocol deviations and receiving one dose of drug.
3.- Response evaluable population: to be included in this population, they must meet the following characteristics:

a. Be eligible, having a measurable disease.
b. Completing the evaluation of all baseline injuries at least once with the same techniques used at baseline.
c. No major protocol violations (such as concomitant anti-cancer treatment).

12.1.2 Population for safety:

Patients evaluable for toxicity: all patients who have received at least one dose of treatment will be included in this population.

12.2 Statistical analysis

12.2.1 Methods

Data will be entered into data entry software guaranteeing information integrity. Software complying with data and software validation guidelines published by the regulatory agencies will be used for this purpose. For this, they must have incorporated an audit trail system ensuring traceability of any change made in data, indicating for each change: the date of change, person making it and reason, as well as the previous data.

Oracle Clinical, meeting the previous requirements, will be used for data capture in this project.
To ensure data quality, control mechanisms will be established including dual data entry, consistency checks of information, and issue of clarifications to the investigators with the inconsistencies detected. These activities will follow the standardized operating procedures, that will include prior preparation of a data management plan, and also a clarification plan with checks to be performed.
Statistical programs will be prepared based on the SAS statistical package. The analysis plan will be completed before the database is closed.
Quantitative variables will be provided using size, mean, standard deviation, two-side 95% confidence interval, median, interquartile range, minimum, and maximum.
Qualitative variables will be summarized in a table that will include absolute and relative frequencies per treatment group and in the whole population. Depending on the variable, two-sided 95% confidence intervals may be provided.

12.2.2 Primary endpoint analysis

Phase I.

To assess the safety profile and established the maximum dose tolerated / recommended dose of sorafenib in combination with gemcitabine and radiation therapy concomitantly.

The MTD is defined as the dose level where ≤ 2 patients experience DLT and the recommended dose (RD) will be the MDT.

Phase II

The percentage of progression-free rate at 6 months will be established with the corresponding 95% confidence intervals for safety. The hypotheses indicated in the determination of sample size will be compared: P(0)=0.30 and P(1)=0.50.
If at least 17 patients were free from progression at 6 months, it can be concluded that the combination is active enough for subsequent development.

12.2.3 Analysis of secondary endpoints

Phase II

- Percent response (PR/CR) by RECIST criteria.
- Overall survival.

- Toxicity occurring using the National Cancer Institute toxicity criteria (NCI CTC) version 3.0.
- Percentage of patients undergoing surgery after the study treatment

In the qualitative parameters, percentage response and toxicity, the confidence intervals will be calculated.
For global and progression-free survival, the Kaplan-Meier curve will be applied, calculating the median with the confidence intervals.

12.2.4 Tolerability analysis

The analysis of tolerability will be performed in the population evaluable for safety.

12.2.5 Treatment exposure

For all patients the duration of follow-up, corresponding to the time (in days) between inclusion and withdrawal from the study, will be calculated.
For patients lost to follow-up, the time of study discontinuation will be the date of the last visit or information.
Treatment duration (in days) will be calculated for each patient as the time from the date of first administration to the treatment discontinuation date.

12.2.6 Adverse events

Adverse events will be coded using the NCIC-CTCAE v3.0 dictionary.

The following will be described in the total population and in the tolerability population during the treatment period:

- Number and proportion of patients who have experienced at least one adverse event
- Number and proportion of patients who have experienced at least one serious adverse event
- Number and proportion of patients who have experienced at least one grade 3/4 adverse event
- Number and proportion of patients who have experienced at least one grade 3/4 adverse event considered by the investigator to be related to treatment
- Number and proportion of patients who have experienced at least one adverse event considered by the investigator to be related to treatment

- Number and proportion of patients who have experienced at least one adverse event whose relationship to treatment has been considered by the investigator to be related or unknown
- Number and proportion of patients withdrawn from the trial due to adverse events

Adverse events will be described by system and will be reported as number of events and number of patients who experienced the adverse event at least once.
Adverse event characteristics (relationship to study treatments, course, seriousness, severity, dose adjustment, etc.) will be described for the overall events occurring during the treatment period.

12.3 Planned interim statistical analyses

No interim analyses are planned.

12.4 Determination of sample size and planned number of subjects to be included

Phase I

A standard dose escalation study will be used, with cohorts of 3-6 patients. At least 3 patients will be treated in each dose level to calculate toxicity. If 1 of 3 patients show DLT, at least 3 patients will be included in this dose level. The MTD is defined as the dose level where ≤ 2 patients experience DLT and the recommended dose (RD) will be the MDT.

Phase II :

The percentage of patients free from progression at 6 months is the primary endpoint of Phase II of the study. The minimum percentage of PFR at 6 months is established at ≤ al 30% (P0) and the optimum PFR is established at ≥ 50% (P1), with an alpha error of 0.05 and a beta error of 0.80. With these assumptions, the sample size will be 39 patients. If at least 17 patients were free from progression at 6 months, it can be concluded that the combination is active enough and must be tested.
The percentage of progression-free rate will be established with the appropriate confidence interval. In addition, the Kaplan-Meier curve for progression-free survival will be obtained, calculating the median with the confidence intervals.

12.5 Significance level

The significance level of the statistical tests used will be 0.05.

12.6 Criteria for study completion

It should be documented if the patient completes the study or not. If for any reason the patient discontinues the treatment, the reason should be entered in the end of treatment CRF. The reasons why a patient can withdraw or be removed from the study will be those established in the end criteria section of this protocol.

12.7 Procedures for justifying missing data

During the study development, clarifications should be given for missing values involved in the analysis of the primary endpoint.
Furthermore, the statistical analysis plan will detail the variables for which missing values and the method used can be estimated.
For the primary endpoint, no estimation of missing data will be performed.

12.8 Procedures for reporting deviations from the original statistical plan

For performing the final report a statistical analysis plan (SAP) will be prepared prior to the closure of the clinical database, that will detail all the analyses, procedures to be performed, and techniques to be used. The SAP will show any change and/or amendment from the originally planned analyses.
In the event that changes should be made in the report in the analyses planned in the SAP, this will be noted in the final report.

13.- REFERENCES

1. Martínez J., Pérez-Mateo M. Tratamiento de las enfermedades gastroenterológicas, Capítulo 46: Tumores del páncreas Publicado en AEG - Asociación Española de Gastroenterología. www.manualgastro.es.
2. Feliú J. ¿Debe recomendarse tratamiento citostático a un enfermo con carcinoma de páncreas metastásico?. Oncología 2004; 27(4): 81-84.
3. Gudjonsson B. Carcinoma of the pancreas: critical análisis of cost results of resection, and the need for standarized reporting. J Am Coll Surg 1995; 181:483-6.
4. Haller DG. New perspectives in the management of pancreas cancer. Semin Oncol 2003; 30 (suppl 11):3-10.
5. Cellini N, Costamagna G, Morganti AG, et al. Concomitant radiochemotherapy in unresectable carcinoma of the exocrine pancreas: cost-effectiveness analysis. Rays 1999; 24:447-52.
6. Willett CG, Czito BG, Bendell JC, et al. Locally advanced pancreatic cancer. *J Clin Oncol* 2005; 23: 4538-4544.
7. Ficha técnica de Gemzar® 2005 Eli Lilly Company.
8. Burris HA III, Moore MJ, Andersen J, et al. Improvements in survival and clinical benefit with gemcitabine as first-line therapy for patients with advanced pancreas cancer: a randomized trial. *J Clin Oncol.* 1997; 15:2403-2413
9. Rothenberg ML, Moore MJ, Cripps MC, Andersen JS, Portenoy RK, Burris HA 3rd, et al. A phase II trial of gemcitabine in patients with 5-FU-refractory pancreas cancer. Ann Oncol 1996; 7:347-53.
10. Lawrence TS, Chang EY, Hahn TM et al. Radiosensitization of pancreatic cancer cells by 2', 2'-difluoro-2'-deoxycytidine. *International Journal of Radiation Oncology, Biology, Physics* 1996; 34(4):867-872.

11. Pauwels B, Korst AE, Pattyn GG et al. Cell cycle effect of gemcitabine and its role in the radiosensitizing mechanism in vitro. *International Journal of Radiation Oncology, Biology, Physics* 2003;57(4):1075-1083.

12. Milas L, Fujii T, Hunter N et al. Enhancement of tumor radioresponse in vivo by gemcitabine. *Cancer Research* 1999;59(1):107-114.

13. Moore AM, Cardenes H, Johnson CS et al: A phase II study of gemcitabine in combination with radiation therapy in patients with localized, unresectable, pancreatic cancer: A Hoosier Oncology Group Trial. *Journal of Clinical Oncology*, 2004 Proc Am Soc Clin Oncol 4105a.

14. Wolff RA, Evans DB, Gravel DM et al. Phase I trial of gemcitabine combined with radiation for the treatment of locally advanced pancreatic adenocarcinoma. *Clinical Cancer Research* 2001; 7(8):2246-2253.

15. Kolch W. Meaningful relationships: the regulation of the Ras/Raf/MEK/ERK pathway by protein interactions. Biochem J 2000; 351:289-305.

16. Monografía Nexavar® 2006 Bayer Pharmaceuticals Corporation.

17. Awada A, hendlisz A, Gil T et al. Phase I safety and pharmacokinetics of BAY 43-9006 administered for 21 Días on/7 Días off in patients with advanced, refractory solid tumours.Br J Cancer. 2005 May 23;92(10):1855-61.

18. Clark JW, Eder JP, Ryan D et al. Safety and pharmacokinetics of the dual action Raf kinase and vascular endothelial growth factor receptor inhibitor, BAY 43-9006, in patients with advanced, refractory solid tumors. Clin Cancer Res. 2005 ;11(15):5472-80.

19. Moore M, Hite HW, Siu L et al. Phase I study to determine the safety and pharmacokinetics of the novel Raf kinase and VEGFR inhibitor BAY 43-9006, administered for 28 Días on/7 Días off in patient with advanced, refractory solid tumors. Ann Oncol. 2005 Oct;16(10):1688-94.

20. Strumberg D, Richly H, Hilger RA et al. Phase I clinical and pharmacokinetic study of the Novel Raf kinase and vascular endothelial growth factor receptor inhibitor BAY 43-9006 in patients with advanced refractory solid tumors. J Clin Oncol. 2005 Feb 10;23(5):965-72.

21. Calhoun, ES, Jones JB, Ashfaq R, et al. BRaf and FBXW7 (CDC4, FBW7, AGO, SEL10)

mutations in distinct subsets of pancreatic cancer. *Am J Pathol* 2003; 163: 1255-1260.

22. Xiong HQ. Molecular targeting therapy for pancreatic cancer. *Cancer Chemother Pharmacol* 2004; 54: S69-S77.

23. Korc M. Pathways for aberrant angiogenesis in pancreatic cancer. *Mol Cancer* 2003; 2:8 .

24. Itakura J, Ishiwata T, Friess H, et al. Enhanced expression of vascular endothelial growth factor in human pancreatic cancer correlates with local disease progression. *Clin Ca Res* 1997; 3:13009-1316.

25. Karayiannakis AJ, Bolanaki H, Syrigos KN, et al. Serum vascular endothelial growth factor levels in pancreatic cancer patients correlate with advanced and metastatic disease and poor prognosis. *Cancer Lett* 2003; 194: 119-124.

26. Baker CH, Solorzano CC, Fidler IJ: Blockade of vascular endothelial growth factor receptor signaling for therapy of metastatic human pancreatic cancer. *Cancer Res* 2002 ; 62:1996-2003.

27. Bruns CJ, Shrader M, Harbison MT, et al. Effect of the vascular endothelial growth factor receptor-2 antibody DC-101 plus gemcitabine on growth, metastasis, and angiogenesis of human pancreatic cancer growing orthotopically in nude mice. *Int J Cancer* 2002; 102; 101-108.

28. Wallace JA, Locker G, Nattam S, Kasza K, Wade-Oliver K, Vokes EE, Kindler HL. Sorafenib (S) plus gemcitabine (G) for advanced pancreatic cancer (PC): A phase II trial of the University of Chicago Phase II Consortium. 2007 Gastrointestinal Cancers Symposium; Abstract No: 137.

29. Li J, Huang S, Armstrong EA, et al. Angiogenesis and radiation response modulation after vascular endothelial growth factor receptor-2 (VEGFR2) blockade, *Int J Radiat Oncol Biol Phys* 2005; 62:1477-1485

30. Gorski DH, Beckett MA, Jaskowiak NT et al Blockage of the vascular endothelial growth factor stress response increases the antitumor effects of ionizing radiation. *Cancer Res* 1999, 59:3374-3378

31. Geng L, Donnely E, McMahon G, et al. Inhibition of vascular endothelial growth factor receptor signaling leads to reversal of tumor resistance to radiotherapy. *Cancer Res* 2001, 61:2413-2419

32. Kozin SV, Boucher Y, Hicklin DJ, et al. Vascular endothelial growth factor receptor-2- blocking

antibody potentiates radiation-induced long-term control of human tumor xenografts. *Cancer Res* 2001; 61: 39-44.

33. Munshi A, Tanaka T, Hobbs M, et al. Radiosensitization of human melanoma cells by sorafenib (BAY 43-9006), a multi-kinase inhibitor. AACR 2006; 4671a.

34. Vincent P, et al. Preclinical chemotherapy with the Raf kinase inhibitor BAY 43-9006 in combination with gefitinib, vinorelbine, gemcitabine or doxorubicin. Proc ASCO 2003

35. Edmund A. Gehan, Mariella C. Tefft . Will There Be Resistance to the RECIST (Response Evaluation Criteria in Solid Tumors). Journal of the National Cancer Institute 2000; 92(3):179-81.

36. P Therasse, SG Arbuck, EA Eisenhauer, J Wanders, RS Kaplan, L Rubinstein, J Verweij, M Van Glabbeke, AT van Oosterom, MC Christian, SG Gwyther. Response Evaluation Criteria in Solid Tumors (RECIST). New Guidelines to Evaluate the Response to Treatment in Solid Tumors. J Natl Cancer Inst 2000; 92:205-216.

14.- ANNEX I. INVESTIGATORS AND PARTICIPATING CENTRES

It is enclosed in the annex document.

15.- *ANNEX II. CLINICAL RESEARCH ETHICS COMMITTEES EVALUATING THE STUDY*

The decisions of the CRECs evaluating this protocol will be given in an enclosed document.

16.- ANNEX III. CASE REPORT FORM

It is enclosed in the annex document.

17.- ANNEX IV INVESTIGATOR'S BROCHURE

It is enclosed in the annex document.

18.- ANNEX V. STANDARD OPERATING PROCEDURES

The standard operating procedures of CRO PIVOTAL will be followed. They are enclosed in the annex document.

19.- ANNEX VI. DEFINITIONS

1. Definitions for the study variables

Progression free rate at 6 months (PFR)

Percentage of patients without radiological disease progression at 6 months from inclusion in the study.

Progression-free survival (PFS):

It is defined as the time from inclusion in the study to radiological disease progression.

Overall Survival time (OST)

It must be measured from the date of inclusion (baseline assessment) to the date of death for any cause.

Best Overall Response (BOR)

It is defined as the best tumoural response recorded during or after 30 days after the end of treatment, which is confirmed according to RECIST criteria.

Objective Response Rate (ORR)

It is defined as the percentage of patients with confirmed complete or partial response.

Disease Control Rate (DCR)

It is defined as the percentage of patients with confirmed complete or partial response or stable disease.

Objective response time (CR;complete or PR;partial)

(ORT)

It is only defined for patients with an objective tumour response and is the time from the date of inclusion (baseline assessment) to the date of the first documented objective tumour response.

Response Duration (RD)

It is defined only for patients that show a confirmed complete or partial objective response, and is the time from the date of the first objective response to the date of confirmed objective disease progression.

2. RECIST (35,36)

Response and progression will be evaluated using the RECIST (Response Evaluation Criteria in Solid Tumors Group). (35,36)

Measurability of lesions

Tumour lesions can be rated as follows: measurable/quantifiable.

(lesions that can be measured appropriately in at least one dimension [note the largest diameter] as > 20 mm with conventional techniques or as > 10 mm with spiral CT [see section 2.2] or non-measurable (all other lesions, including small lesions [largest diameter < 20 mm with conventional techniques or < 10 mm with spiral CT] and really non-measurable lesions).

The lesions considered as really non-measurable are as follows: bone lesions, leptomeningeal disease, ascites, pleural/pericardial effusion, inflammatory disease of the breast, lymphangitis cutis/pulmonis, abdominal masses not confirmed and followed by imaging diagnosis techniques, and cyst lesions.

Measuring methods

To identify and inform each lesion, the same assessment method and the same technique should be used at baseline and during the follow-up. When diagnostic imaging methods and clinical examination have been used for evaluating the anti-tumoural effect of a treatment, the former will be preferred.

- Clinical examination. Clinically identified lesions will be only considered measurable if they are superficial (for instance, palpable skin nodes and lymph nodes). In the case of skin lesions, it is recommended to document them by colour pictures that show a rule for estimating the size of the lesions.
- Chest X-ray. The lesions that are evident in the chest X-ray are accepted as measurable lesions when they are clearly defined and surrounded by aired lung. however, the CT is preferred.
- CT and MRI. CT and MRI are the best, most reproducible methods available today for

measuring target lesions chosen for response assessment. Both the conventional CT and the MRI should be performed with adjacent sections of 10 mm or less in thickness. With regard to spiral CT, it should be perform with a rebuilding algorithm with 5 mm adjacent sections; this specification is valid for chest, abdomen and pelvis tumours, while tumours of the had and neck and of the extremities usually require specific protocols.

- Ultrasonography. When the primary study endpoint is the assessment of the objective response, ultrasonography should not be used to measure tumoural lesions that are not clinically easily accessible. However, it can be used as an alternative to clinical measurements in the case of lymph nodes, subcutaneous lesions and palpable superficial thyroid nodes. Ultrasonography may be also valuable for confirming the complete disappearance of superficial lesions evaluated usually by clinical examination.
- Endoscopy and laparoscopy. The use of these techniques for objective tumour assessment has not been validated completely or extensively yet. Their use in this specific setting requires a sophisticated equipment and a high level of experience, only available at some centres. Therefore, the use of these techniques for objective tumour response should be limited to validation purposes at specialised centres. However, these techniques can be useful for confirming a complete histopathological response when biopsy samples are obtained.
- Tumoural markers. Tumour markers cannot be used alone for response assessment. However, if tumour markers were initially above the upper normal limit, they should return to normal limits for a patient to be considered in complete clinical response when all tumour lesions have disappeared. Specific additional criteria for the standardised use of specific prostate antigen and CA 25 are currently under validation to support clinical trials.
- Cytology and histology. In rare cases (for instance, after treatment, to distinguish between residual benign lesions and residual malignant lesions in tumour types such as stem cell tumours) cytology and histology techniques can be used to distinguish between partial response and complete response. When the measurable tumour meets the criteria of response or stable disease, in case of effusion appearing or worsening during the treatment, cytological confirmation of its nature is required. In these cases, the cytology study of the fluid obtained will allow for distinguishing between response or stable disease (an effusion can be a secondary effect of the treatment) and progressive disease (if the neoplastic nature of the fluid is confirmed). The new techniques to establish better the objective tumour response will be integrated in these criteria when they have been validated adequately for use in the setting of the evaluation of tumour response.

Baseline documentation of lesions

All measurable lesions, up to 5 lesions per organ and a total of 10 lesions, representative of all organs affected, should be identified as target lesions and registered and measured at baseline. The target lesions will be chosen by size (those with the greatest diameter will be chosen) and their suitability for repeated, accurate measures (by imaging techniques or clinically). The sum of the largest diameters of all target lesions will be obtained, which will be reported as the sum of the largest diameters at baseline. This sum of the largest diameters at baseline will be used as reference for characterising the objective tumour response.

All the other lesions (or disease areas) will be identified as non-target lesions and will be also recorded at baseline. Although measuring these lesions is not necessary, during the follow-up the presence or absence of each of them should be noted.

Response criteria for the target lesions

- Complete response: disappearance of all target lesions.
- Partial response: reduction of at least 30% in the sum of the largest diameter of the target lesions, taking as reference the sum of the largest diameters at baseline.
- Progressive disease: increase of at least 20% in the sum of the largest diameter of the target lesions, taking as reference the lowest value of the sum of all largest diameters recorded from the start of treatment or the appearance of one or more new lesions.

Stable disease: neither a reduction enough to rate the case as partial response nor an increase enough to rate it as progressing disease, taking as reference the lowest value of the sum of the largest diameter recorded from the start of treatment.

Assessment of non-target lesions

- Complete response – disappearance of all non-target lesions and normalisation of tumour marker levels.
- Incomplete response / stable disease – persistence of one or more non-target lesion(s) or maintenance of the level of tumour markers above normal ranges.
- Progressive disease – appearance of one or more new lesions or unequivocal progression of existing non-target lesions.

Global responses for all possible combinations of tumour response in target and non-target lesions, with or without appearance of new lesions

Target Lesions	Non-Target Lesions	New lesions	Overall Response
CR	CR	No	CR
CR	Incomplete response / SD	No	PR
PR	Non-PD	No	PR
SD	Non-PD	No	SD
PD	Any	Yes or No	PD
Any	PD	Yes or No	PD

CR = complete response; PR = partial response; SD = stable disease; and PD = progressive disease. For more information, see the text.

3. ECOG performance status

0	Active Can fulfil the same activity as before the diagnosis.
I	Some restriction in activities. Symptomatic. Ambulatory and able to fulfil sedentary work.
II	Symptomatic. Stays in bed or sitting less than 50% of waking time.
III	Symptomatic. Stays in bed or sitting less than 50% of waking time.
IV	Bedridden 100% of waking time.

4. New York Heart Association functional criteria

Class I	No limitation for regular physical activity.
Class II	Minor limitation to regular physical activity with fatigue, palpitations, dyspnea or angina pectoris.
Class III	Less physical activity than usual causes minor limitation with fatigue, palpitations, dyspnea or angina pectoris, but does not appear at rest.
Class IV	The symptoms of heart failure or angina pectoris can occur even at rest, worsening with any physical activity.

5. TNM classification

Tumour (T)

- T0 No evidence of primary tumour
- Tis Carcinoma in situ
- T1 Tumour limited to the pancreas ≤ 2 cm
- T2 Tumour limited to the pancreas > 2 cm
- T3 Tumour extent out of the pancreas without involving the celiac trunk or the upper mesenteric artery

- T4 Tumour extension involving the celiac trunk or the upper mesenteric artery (unresectable tumour)

Regional lymph nodes (N)

- N0 No involvement of local-regional nodes.
- N1 Involvement of local-regional nodes.

Distant metastasis (M):

- M0 No distant metastasis
- M1 Distant metastasis

Staging

- **Stage 0** Tis, N0, M0
- **Stage IA** T1, N0, M0
- **Stage IB** T2, N0, M0
- **Stage IIA** T3, N0, M0
- **Stage IIB** T1-3, N1, M0
- **Stage III** T4, Any N, M0
- **Stage IV** Any T, any N, M1

20.- *ANNEX VII. DECLARATION OF HELSINKI*

The investigator should perform the study in compliance with the guidelines of the Declaration of Helsinki. The copies of the Declaration of Helsinki and subsequent amendments will be provided under express request or can be obtained through the webpage of the World Medical Association at http://www.wma.net/e/policy/b3.htm.

21.- ANNEX VIII. SAE REPORT FORM

The form will be enclosed.

22.- ANNEX IX. INSTRUCTIONS FOR PERFORMING THE CT.

1.- Previous conditions of the patient:

- Absence of allergy to iodinated contrasts.
- Renal function which allows for performing CT with contrast.
- Absence of surgery of the pancreas in the past 30 days.
- Absence of bile prostheses.

2.- Technique demandable:

For the initial inclusion study:

i. Multidetector CT (preferable) or spiral CT
ii. Obtaining images with collimation and increase of 5 mm or less.
iii. Dynamic study in pancreatographic and portal venous phases.

For follow-up studies:

Multidetector CT (preferable) or spiral CT
Obtaining images with collimation and increase of 5 mm or less.
Dynamic study in portal venous phase. The pancreatographic phase is desirable, though in the follow-up could be optional to prevent exclusion of patients for not having a follow-up CT deviating from the protocol.

3.- Criteria of inoperability by CT:

Arterial involvement:

Celiac trunk, hepatic artery, upper mesenteric artery. The tumour contacts focally with the artery in 180º or more of the vessel section.

Venous involvement:

Portal vein, confluent portomesenteric or upper mesenteric vein. Any contact between the vein and the tumour in 180º or more of the vessel section; teardrop sign; marked dilation of the gastrocolic venous trunk. (If the extent of venous involvement is below 2 cm in the longitudinal axis of the VMS, some surgical groups can consider that the patient is operable).

Distant metastasis:

Liver, peritoneum, paraaortic adenopathies, lung. Peripancreatic adenopathies are not a contraindication to surgery and, therefore, are not an exclusion criterion.

4.- Data to be contained in the radiology report and to be entered in the CRF

The radiology report must contain, in the inclusion studies, the maximum tumour size in the axial plan and the information on the infiltration of all structures involving inoperability, applying the above criteria. The follow-up studies must specify greatest tumour size. The data sheet will contain a chart including the data from the radiology report.

TEST ITEM	YES	NO
Does the CT study protocol follow the proposed protocol?		
Distant metastasis (liver, lung carcinomatosis, retroperitoneal adenopathies)		
Infiltration of adjacent viscera (stomach, colon, ID)		
Infiltration of CT		
Infiltration of AH		
Infiltration of AMS		
VP infiltration		
Infiltration of VMS involving inoperability		

Schima W, Ba-Ssalamah A, Kölblinger C, Kulinna-Cosentini C, Puespoek A, Götzinger P. Pancreatic adenocarcinoma. Eur Radiol 2007;17:638–649.
Li H, Zeng MS, Zhou KR, Jin DY, Lou WH. Pancreatic adenocarcinoma. The different CT criteria for peripancreatic major arterial and venous invasion. J Comput Assist Tomogr 2005;29:170–175.

Lu DSK, Reber HA, Krasny RM, Kadell BM, Sayre J. Local staging of pancreatic cancer: criteria for unresectability of major vessels as revealed by pancreatic-phase thin section helical CT. AJR Am J Roentgenol 1997;168:1439–1443.
O'Malley ME, Boland GWL, Wood BJ, Fernandez-del Castillo C, Warshaw AL, Mueller PR Adenocarcinoma of the head of the pancreas: determination of surgical unresectability with thin-section pancreatic-phase helical CT. AJR Am J Roentgenol 1999;173:1513–1518.

5.- Response assessment:

The RECIST criteria will be applied:

Maximum dimension of the tumour in the axial plan in mm, in each study.

Complete response:
Disappearance of the lesion

Partial response:
Reduction of 30% from the baseline tumour size

Stable disease:
Reduction of maximum diameter > 30 % or increase < to 20% of the baseline size.

Progressive disease:
Increase in the maximum diameter > 20% of lowest tumour size (in baseline CT or in any follow-up) from patient inclusion. Any new lesion considered to be tumoural.

23.- ANNEX X. COMMITMENT OF THE INVESTIGATORS

Dr. ________________________ of the centre ___________________________________

Certifies:

That he knows and agrees to participate as principal investigator in the clinical trial entitled:
A PHASE I-II MULTICENTER, NON-RANDOMISED CLINICAL TRIAL ON THE SAFETY AND EFFICACY OF THE COMBINATION OF SORAFENIB (BAY 43-9006), GEMCITABINE AND RADIATION THERAPY CONCOMITANTLY IN THE TREATMENT OF PATIENTS WITH LOCALLY ADVANCED ADENOCARCINOMA OF THE PANCREAS
Sponsor code: GEMCAD01-07
With EudraCT code: 2007-003211-31

And that the investigational product is: Sorafenib (BAY 43-9006)

That he undertakes that each subject is treated and monitored following the provisions of the protocol authorised by the Clinical Research Ethics Committee and by the Spanish Agency for Medicinal Products and Medical Devices.

That it will abide by the ethical rules applicable to this type of studies.

That this study will be performed with the collaboration of

....

....

....

as collaborating investigators.

In ____________________, on _______________ ________________ 200__.

Signed:
